# Biomimetic Ion Channel Design for Simultaneous Lithium‐Ion Flux Regulation and Interfacial Stabilization in Lithium Metal Batteries

**DOI:** 10.1002/smll.74056

**Published:** 2026-06-03

**Authors:** Qian Cheng, Ke Fan, Jun‐Ming Cao, Zezhou Lin, Jiamin Fu, Shengjie Xia, Chao Wang, Yao Liu, Simeng Zhang, Changhong Wang, Xueliang Sun, Haitao Huang

**Affiliations:** ^1^ Department of Applied Physics The Hong Kong Polytechnic University Hong Kong China; ^2^ Eastern Institute For Advanced Study Ningbo Institute of Digital Twin, Eastern Institute of Technology Ningbo China; ^3^ Zhejiang Key Laboratory of All‐Solid‐State Battery Ningbo Key Laboratory of All‐Solid‐State Battery Ningbo China; ^4^ School of Materials Science and Engineering Anhui University Hefei China; ^5^ Department of Mechanical and Materials Engineering University of Western Ontario London Ontario Canada; ^6^ Research Institute For Advanced Manufacturing The Hong Kong Polytechnic University Hong Kong China

**Keywords:** bionic design, crown ether, interfacial chemistry, Li^+^ flux regulation, lithium metal batteries, separator modification

## Abstract

Lithium metal batteries hold great promise for future energy storage due to their high energy density and potential for fast charging, making them ideal for applications in electric vehicles and portable electronics. However, lithium metal batteries usually suffer from rapid performance degradation because of the unstable electrode/electrolyte interface. To address this, we integrate bionic ion channels into commercial battery separators. This structure features metal–organic framework (MOF)‐encapsulated benzo‐12‐crown‐4‐ether, mimicking biological ion channels. It enables rapid Li^+^ transport and uniform flux distribution, which suppress lithium dendrite growth. The crown ether sites in bionic ion channels weaken Li^+^‐solvent coordination, forming an anion‐rich solvation sheath. These anions preferentially decompose, generating a passivation layer at the anode interface that is rich in inorganic LiF and Li_3_N. Consequently, Li||Li symmetric cells achieve stable plating/stripping for over 1500 h, and LiFePO_4_||Li full cells retain 86% capacity after 1200 cycles. This work provides a promising strategy for synergistic regulation of Li^+^ flux and interfacial chemistry through bionic design.

## Graphical abstract

Inspired by biological cell membranes, BCE‐encapsulated MOFs create bionic Li^+^ transport channels. These channels homogenize Li^+^ flux via Li^+^‐crown ether ion‐dipole interactions and manipulate solvation structures to form inorganic‐rich SEI layers. This synergy enables dendrite‐free Li deposition, achieving 1500 h stability in Li||Li cells and 86% capacity retention after 1200 cycles in Li||LiFePO_4_ cells at 2C.

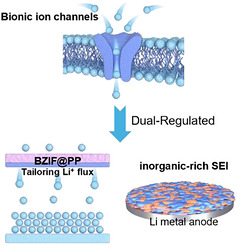

## 1 Introduction

The urgent need for high‐energy‐density battery systems is driven by the increasing demand for efficient energy storage solutions across various sectors, including electric transportation, portable electronics, and grid‐scale energy storage systems. Li metal has been widely explored as a promising candidate for high‐energy‐density battery systems due to its high theoretical specific capacity (3860 mAh g^−1^), the lowest electrochemical potential (−3.04 V vs the standard hydrogen electrode), and low density (0.53 g cm^−3^) [1, 2]. However, the interface issues of lithium metal anode, such as lithium dendrites [3, 4] and unstable solid electrolyte interphase (SEI) [5, 6, 7], are the bottlenecks restricting the electrochemical performance of lithium metal batteries (LMBs).

In recent years, a lot of research work has been devoted to addressing these interfacial challenges by designing and optimizing electrodes [8, 9, 10], electrolytes [11, 12, 13], and their interfaces [14, 15, 16, 17]. One critical aspect of this optimization is the transportation of lithium ions (Li^+^) through various components of the battery system. Understanding the pathways and mechanisms of Li^+^ transport is essential for enhancing battery performance. Factors such as ion concentration gradients [18, 19, 20, 21], electric fields [22, 23, 24], and the physicochemical properties of the separator and SEI layer significantly influence Li^+^ flux [25, 26, 27, 28]. The lithium deposition behavior is greatly determined by Li^+^ flux, which includes the diffusion of lithium ions through the separator and SEI layer [29]. The inherent pore heterogeneity of polypropylene (PP) separators leads to uneven distribution of Li^+^ flux in the electrolyte, which is difficult to be completely solved by electrolyte engineering and artificial SEI layer [30, 31]. Consequently, separator modification emerges as an effective strategy for enhancing the electrochemical performance of LMBs. So far, research on separator modification has made some progress, such as mesoporous silica nanospheres coated PP separators [32], covalent organic framework (COF) modified separators [33], PVDF‐based cation‐selective separators [26], crown ether functionalized separators [14], and so on. While these studies have shown promise in mitigating Li dendrites, they have rarely involved regulating the solvation structure to change the composition and structure of the SEI layer on the lithium anode. The SEI layer, as a protective barrier, can avoid direct contact between the lithium metal anode and electrolytes, thus reducing the risk of undesirable side reactions [34]. An inorganic‐rich SEI layer has been widely demonstrated to help enhance the long‐term cycling stability of lithium metal anode [35, 36]. Therefore, designing a separator modification layer that can both suppress lithium dendrites and regulate interfacial chemistry through a scalable approach is of great significance for improving the electrochemical performance of lithium metal anode, while it is still challenging at present.

**FIGURE 1:**
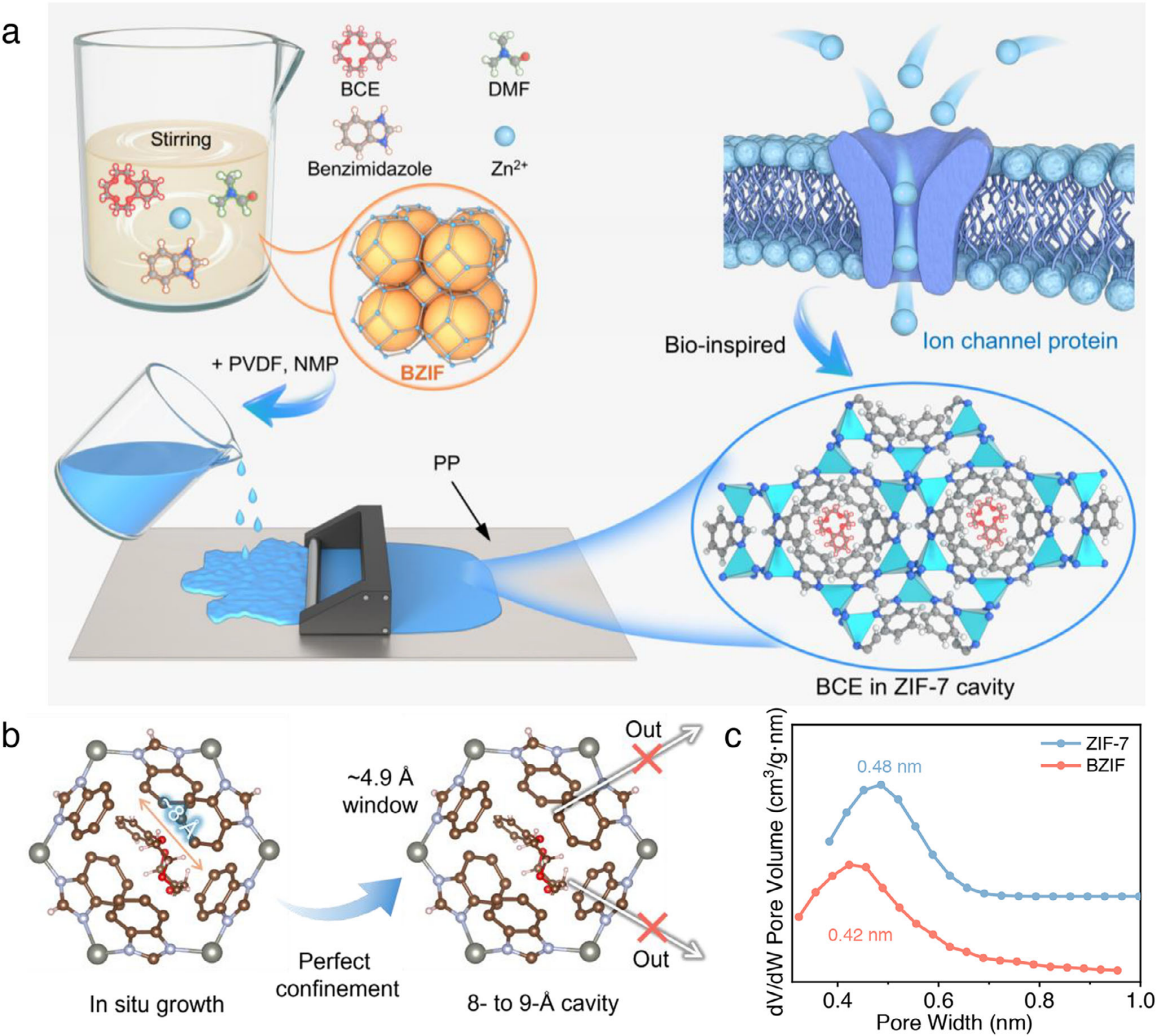
(a) Scheme of the fabrication of BZIF@PP separators and bionic structure of BZIF. (b) Perfect confinement of BCE (∼8 Å) in the ZIF‐7 cavity. (c) Pore size distribution curves of ZIF‐7 and BZIF particles.

Recently, nature‐inspired bionic technologies have been increasingly applied to the modifications of electrodes and electrolytes, which can enhance the electrochemical performance, safety, and sustainability of batteries [37, 38]. Among various biological structures, ion channel proteins of biological cell membranes play a crucial role in regulating mass transfer. For example, some ion channels can achieve selective transport of Na^+^/K^+^ (Figure 1a), which is critical for various metabolic processes and cellular functions [39]. We therefore hypothesize that mimicking such channels in battery separators could provide precise control of Li^+^ transport.

In this work, inspired by biological ion channels, we designed a bionic ion transport channel for polypropylene (PP) separators in LMBs. Encapsulating benzo‐12‐crown‐4‐ether (BCE) molecules within metal‐organic frameworks (MOFs) constitutes this bionic fast lithium‐ion transport channel, enabling uniform Li^+^ flux through the ion‐dipole interaction between Li^+^ and crown ether sites. In addition, these bionic ion channels can manipulate the interfacial solvation structure to induce the formation of an inorganic‐rich SEI layer on the lithium anode. This synergy greatly improves the Coulombic efficiency (CE) and long‐term cycling stability. Considering the above advantages, the bionic ion channels result in a dendrite‐free Li deposition and a stable lithium metal anode interface. The assembled Li||Li symmetrical cells can operate stably up to 1500 h at a current density of 1 mA cm^−2^ without short circuit. Paired with LiFePO_4_ cathodes, full cells exhibit excellent cycle stability (capacity retention of 86% at 2C after 1200 cycles) and rate performance (105.35 mAh g^−1^ at a high rate of 5C).

## 2 Results and Discussion

We synthesized the BCE@ZIF‐7 (BZIF) with biomimetic ion channels by first complexing zinc ions (Zn^2+^) with benzo‐12‐crown‐4‐ether (BCE), followed by benzimidazole addition under stirring, which makes BCE successfully encapsulated into ZIF‐7 cavity in the fabrication (Figure 1a). The cavity of ZIF‐7 is formed by a zinc ring plane perpendicular to a 3‐fold rotoinversion axis, with benzimidazolate linkers oriented parallel to the axis to create a rhombohedral cage (∼8 to 9 Å) [40]. This cage size is well‐matched to the molecular dimension of BCE (∼8 Å), enabling the perfect confinement of a single BCE molecule. After being incorporated in the ZIF‐7 cavity, BCE is unlikely to escape due to the end‐capping by the smaller MOF window (∼4.9 Å) (Figure 1b) [41].

X‐ray diffraction (XRD) pattern of BZIF is similar to that of ZIF‐7 (Figure S1), suggesting that the introduction of BCE molecules during the crystallization process of ZIF‐7 does not disrupt the original crystal structure of ZIF‐7. In addition, scanning electron microscopy (SEM) images in Figure S2 show that both ZIF‐7 and BZIF samples present the morphology of nanoparticles with a size of 100–300 nm. These BZIF nanoparticles form a uniform coating on the PP separator surface, as confirmed by SEM and corresponding elemental mapping of C, N, O, and Zn (Figure S3). The pore size distribution of BZIF, derived from CO_2_ adsorption/desorption isotherms, shows a reduced pore window size compared to pristine ZIF‐7. This decrease confirms that BCE molecules are trapped within the ZIF‐7 cavities during the MOF formation and crystallization process (Figure 1c and Figure S4). Furthermore, the host‐guest interaction between ZIF‐7 and BCE was verified by Fourier transform infrared (FT‐IR) spectroscopy. Characteristic C–O–C stretching vibrations of BCE (originally at ∼1051 and 1220 cm^−1^) show a clear blue shift upon incorporation into ZIF‐7. A concomitant shift is also observed for the Zn‐N stretching vibration (from 1006 to 1007 cm^−1^), indicating a molecular‐level interaction (Figure S5a). Density functional theory (DFT) calculations support this, yielding a favorable binding energy of −1.50 eV between ZIF‐7 and BCE, confirming the thermodynamic stability of the composite (Figure S5b).

**FIGURE 2:**
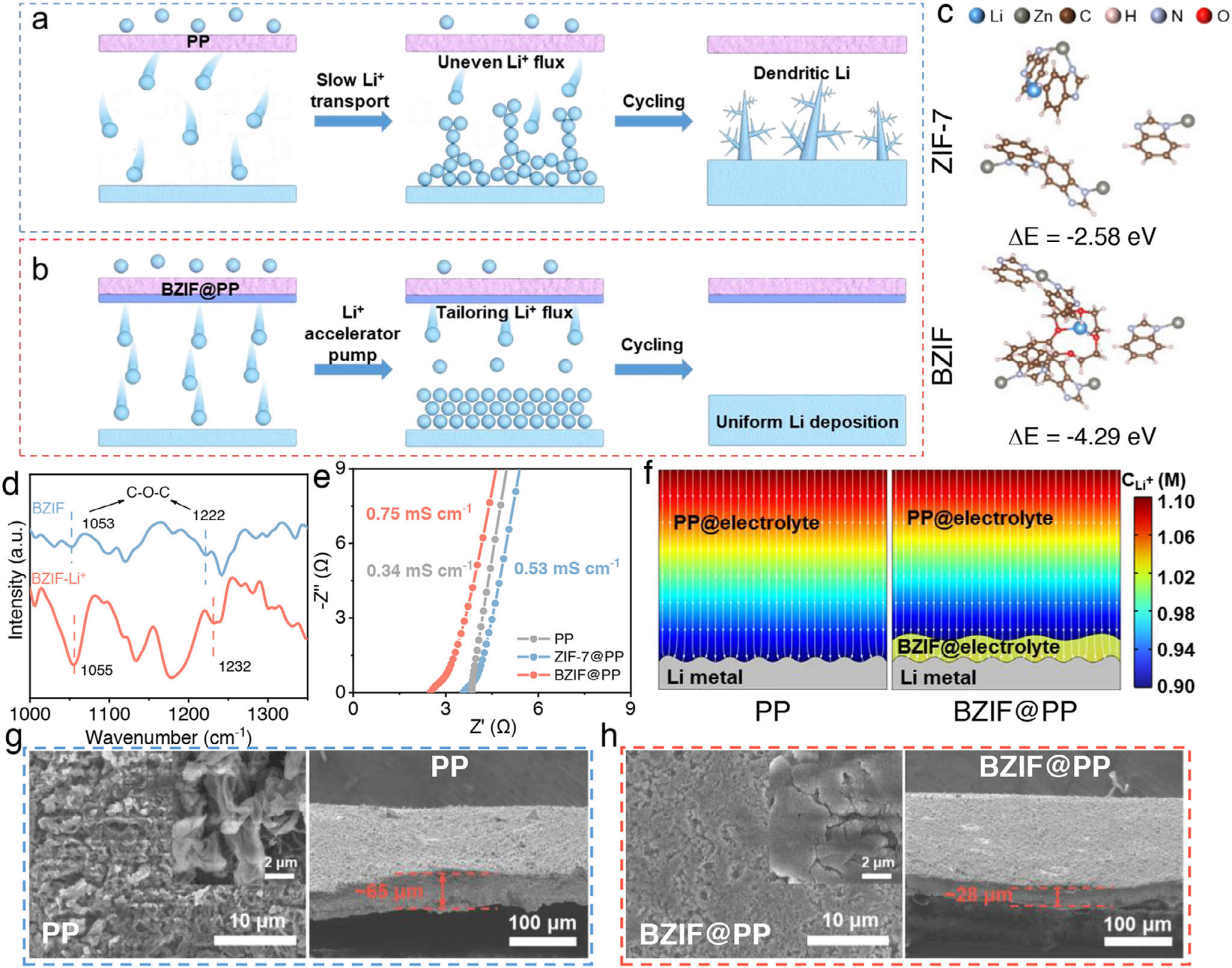
Schematic diagrams of lithium deposition behaviors with (a) PP and (b) BZIF@PP separators. (c) The adsorption energy of Li^+^ for ZIF‐7 and BZIF. (d) FT‐IR spectra of BZIF and BZIF interacting with electrolytes. (e) Nyquist curves of stainless steel (SS)||stainless steel (SS) symmetric cells with PP, ZIF‐7@PP and BZIF@PP separators. (f) COMSOL simulations of Li^+^ concentration distributions from bulk electrolytes to Li anode surface for PP and BZIF@PP separators. SEM images (top view and cross‐section view) of Li deposit with (g) PP and (h) BZIF@PP separators after Li plating on copper foil at a current density of 1 mA cm^−2^ with a plating capacity of 3 mAh cm^−2^.

The schematic diagram of BZIF bionic ion channels regulating Li^+^ flux is shown in Figure 2a,b. Li^+^ tends to distribute randomly through bare PP separators, and the uneven Li^+^ flux on the surface of the lithium anode leads to the growth of lithium dendrites. Conversely, the biomimetic ion channels uniformly redistribute Li^+^ flux by lithiophilic crown ether sites, facilitating a dendrite‐free lithium plating/stripping process. The lithium deposition of Li||Cu half cells in the initial cycle under a current density of 1 mA cm^−2^ with a capacity of 1 mAh cm^−2^ was investigated (Figure S6), where the nucleation overpotential of cells with BZIF ion channels is 36.3 mV, significantly lower than that of PP (58.0 mV) and ZIF‐7@PP (43.1 mV). This reduced nucleation barrier is attributed to the lithiophilic crown ether sites of bionic ion channels.

Further DFT calculations were conducted to confirm the role of the crown ether sites in BZIF for homogenizing Li^+^ flux. As shown in Figure 2c, the adsorption energy of Li^+^ for the BZIF ion channels is −4.29 eV, larger than that of ZIF‐7 (−2.58 eV), which indicates the lithium affinity with the crown ether sites of BZIF. This facilitates uniform Li^+^ transport, which can help reduce Li dendrite formation. FT‐IR spectra were also employed to confirm the interaction of Li^+^ with crown ether sites of BZIF (Figure 2d). The characteristic peaks of C─O─C in BZIF are observed at 1053 and 1222 cm^−1^, which shift to 1055 and 1232 cm^−1^ upon binding with Li^+^. The blue shift of these characteristic peaks confirms the ion‐dipole interaction between Li^+^ and BZIF ion channels.

Li^+^ transport capacity depends critically on ionic conductivity and Li^+^ transference number (*t*_Li_^+^). As shown in Figure 2e, the ionic conductivities of batteries using different separators were tested. The results show that cells employing the BZIF@PP separator achieve the highest ionic conductivity (0.75 mS cm^−1^), exceeding the values for PP (0.34 mS cm^−1^) and ZIF‐7@PP (0.53 mS cm^−1^) separators. Critically, the *t*_Li_^+^ increases from 0.38 (PP) to 0.69 (BZIF@PP) (Figure S7), which is conducive to reducing concentration polarization overpotential. In addition, the Li^+^ distributions from the bulk electrolyte to the anode surface were simulated by the finite element method (Figure 2f). The simulation results reveal that PP separators exhibit a severe Li^+^ concentration gradient between bulk electrolytes and the anode, inducing irregular Li^+^ flux distribution and lithium dendrite growth. In contrast, the BZIF bionic ion channels can accelerate the Li^+^ diffusion kinetics, mitigating interfacial Li^+^ concentration gradient to homogenize Li deposition (Figure S8). SEM images of lithium metal deposited on copper foil were employed to further confirm the ability of bionic ion channels to inhibit lithium dendrites. As shown in Figure 2g, the deposited lithium with PP separators exhibits a rough, uneven, and dendritic morphology under a current density of 1 mA cm^−2^ with a plating capacity of 3 mAh cm^−2^. The corresponding cross‐section Figure also validates a porous and loose structure of lithium deposits with a thickness of 65 µm. In contrast, the deposited lithium metal with BZIF ion channels shows a smooth and uniform bulk lithium morphology with a decreased thickness of 28 µm (Figure 2h), which demonstrates a denser structure of the deposited lithium.

**FIGURE 3:**
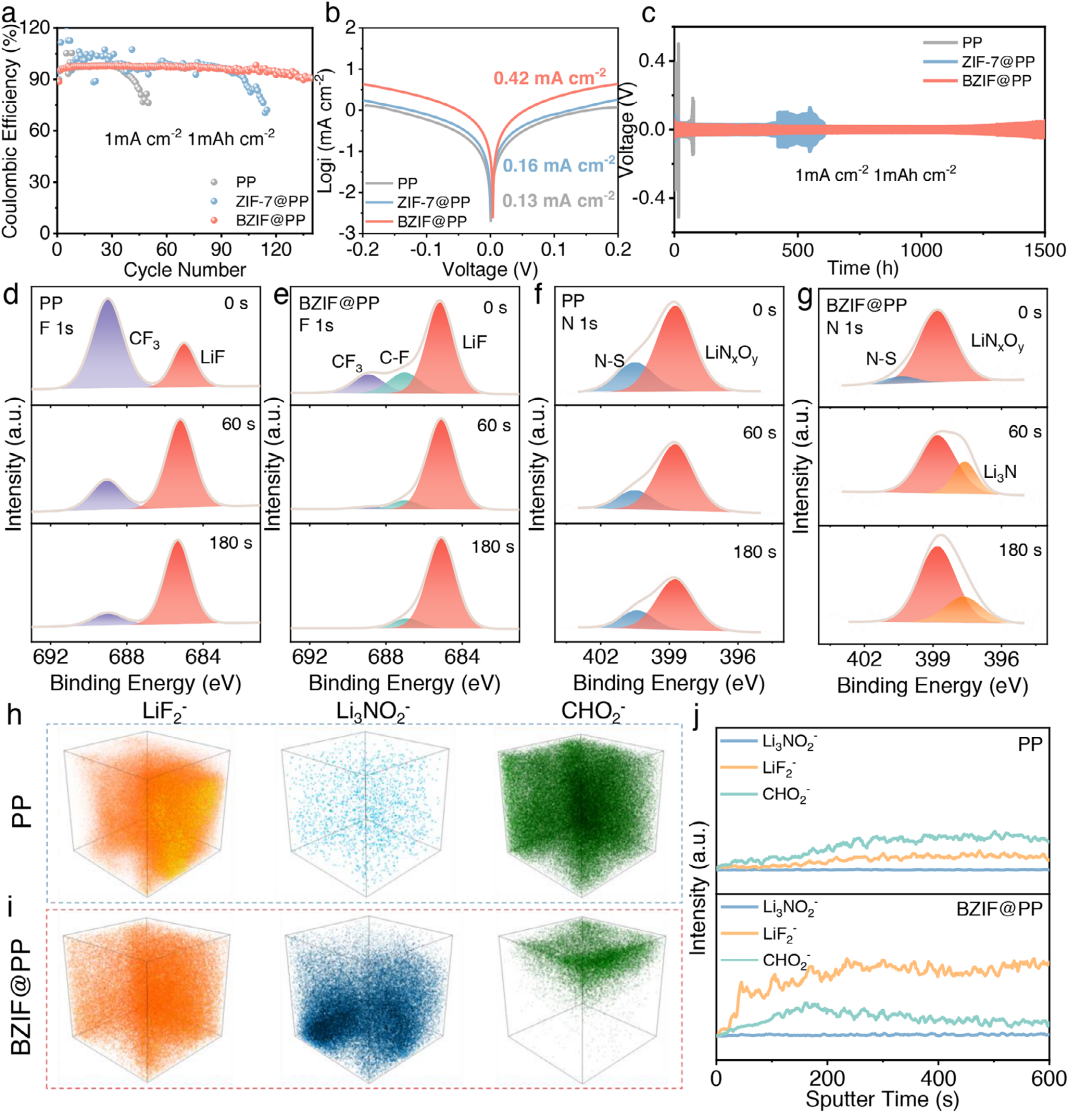
(a) CE test of Li||Cu half cells at a current density of 1 mA cm^−2^ with 1 mAh cm^−2^ area capacity. (b) Tafel plots for Li deposition/stripping of the Li||Li symmetric cells. (c) Long‐term cycling performance of the Li||Li symmetric cells under a current density of 1 mA cm^−2^ with 1 mAh cm^−2^ capacity. The XPS depth profiles of F 1s with (d) bare PP separators and (e) BZIF@PP separators for the cycled Li anode. The XPS depth profiles of N 1s with (f) bare PP separators and (g) BZIF@PP separators for cycled Li anode. TOF‐SIMS 3D spectra of LiF_2_^−^, Li_3_NO_2_^−^, and CHO_2_^−^ fragments for cycled Li anode with (h) bare PP separators and (i) BZIF@PP separators. (j) The corresponding TOF‐SIMS depth profiles for the cycled Li anode with PP and BZIF@PP separators.

The Coulombic efficiency (CE) of Li||Cu half cells was measured to confirm the reversibility of lithium plating/stripping (Figure 3a). The CE of cells with PP separators drops rapidly during long‐term cycling, while those using ZIF‐7@PP exhibit relatively longer lifespans but also fail at the 80th cycle. In contrast, the cells with BZIF ion channels sustain >90% CE for over 140 cycles. Furthermore, the Li^+^ transfer kinetics at interfaces, quantified by exchange current density (*j_0_*) [42], show marked enhancement. Li||Li symmetric cells with BZIF ion channels exhibit an exchange current density of 0.42 mA cm^−2^, about 3.2 times and 2.6 times larger than those of PP (0.13 mA cm^−2^) and ZIF‐7@PP (0.16 mA cm^−2^) (Figure 3b), confirming the accelerated charge transfer kinetics by bionic ion channels. Long‐term Li||Li symmetric cycling (1 mA cm^−2^, 1 mAh cm^−2^; Figure 3c) shows large polarizations and a short cycling life of 100 h and 600 h for PP and ZIF‐7@PP separators, respectively. In sharp comparison, the Li symmetric cells with BZIF bionic ion channels exhibit a stable operation of 1500 h. In the selected regions of long cycling test (Figure S9), the Li||Li cells with BZIF ion channels show a flat overpotential curve with a small overpotential (∼20 mV), indicating a more stable lithium plating and stripping process. This enhanced reversibility of lithium deposition/stripping arises from the lithiophilic crown ether sites of BZIF bionic ion channels, which can effectively reduce the polarization potential [43]. In addition, electrochemical impedance spectroscopy (EIS) measurements of Li||Li symmetric cells show a lower interfacial impedance of cells with BZIF ion channels before and after cycling, suggesting the excellent interfacial stability achieved by biomimetic ion channels (Figure S10). Furthermore, BZIF ion channels can still maintain robust mechanical adhesion and chemical stability after repeated cycling, with no detectable leaching of BCE (Figure S11).

Since the composition and structure of the SEI greatly affect the electrochemical performance of LMBs, additional interface characterization techniques have been employed to investigate the effect of bionic ion channels on SEI. Among these techniques, depth profiling of x‐ray photoelectron spectroscopy (XPS) is particularly effective for analyzing the chemical composition of the SEI. In Figure 3d, the F 1s spectrum with PP separators exhibits two main peaks at 685.2 eV and 688.9 eV, corresponding to LiF and CF_3_ species, respectively. In contrast, the SEI formed with BZIF@PP displays a new peak associated with the C‐F component (Figure 3e). This peak may be attributed to the decomposition of the TFSI^−^ anion promoted by BZIF ion channels [35]. The distribution of surface fluorine species differs significantly between the two separators. The surface of the lithium anode with PP separators is primarily dominated by organic fluorine species, whereas the BZIF@PP exhibits a higher inorganic LiF content on the anode surface. In addition, the ratio of LiF to organic fluorine species remains consistently higher with BZIF ion channels throughout the entire test depth (Figure S12), indicating a LiF‐dominant composition in the SEI layer. In the N 1s spectrum, two peaks at 398.7 eV and 400.5 eV correspond to LiN_x_O_y_ and S‐N species, respectively (Figure 3f). The presence of LiN_x_O_y_ is more pronounced on the lithium anode surface when using BZIF@PP. As sputtering depth increases, Li_3_N appears in the SEI film with BZIF ion channels (Figure 3g), while the N‐S peak diminishes. In contrast, the PP separator does not show a detectable Li_3_N peak, and the N‐S component remains present. This indicates that the BZIF bionic ion channels facilitate deeper decomposition of LiNO_3_, as evidenced by the CV results shown in Figure S13. Such an inorganic‐rich SEI correlates with an anion‐abundant solvation structure, promoting anion decomposition and the formation of inorganic components. For the C 1s spectrum (Figure S14), four peaks at 284.8 eV, 286.5 eV, 288.1 eV, and 290.0 eV correspond to C─C, C─O, C = O, and Li_2_CO_3_, respectively, in the SEI formed with BZIF ion channels. Additionally, an extra peak at 285.8 eV related to the C─N component is observed for PP separators, indicating a more complex composition. This spectrum further demonstrates that the organic content of the SEI layer with BZIF@PP is lower than that with PP separators, which is consistent with the results of the aforementioned F and N spectra. These abundant inorganic species can enhance Li^+^ diffusion in the SEI and inhibit the growth of lithium dendrites. The results from time‐of‐flight secondary ion mass spectrometry (TOF‐SIMS) align well with the XPS analysis, both confirming the formation of an inorganic‐rich SEI induced by the biomimetic ion channels. In Figure 3h,i, the TOF‐SIMS 3D spectra show that the SEI layer associated with BZIF ion channels contains a higher concentration of inorganic fragments, such as LiF_2_^−^ and Li_3_NO_2_^−^. In contrast, the organic fragment CHO_2_^−^ is highly concentrated in the SEI formed with PP separators, as confirmed by TOF‐SIMS depth profiles (Figure 3j and Figure S15). These intrinsic advantages of the SEI formed with bionic ion channels promote uniform lithium deposition and facilitate rapid Li^+^ transport at the interface [44], resulting in a stable anode interface during long‐term cycling.

**FIGURE 4:**
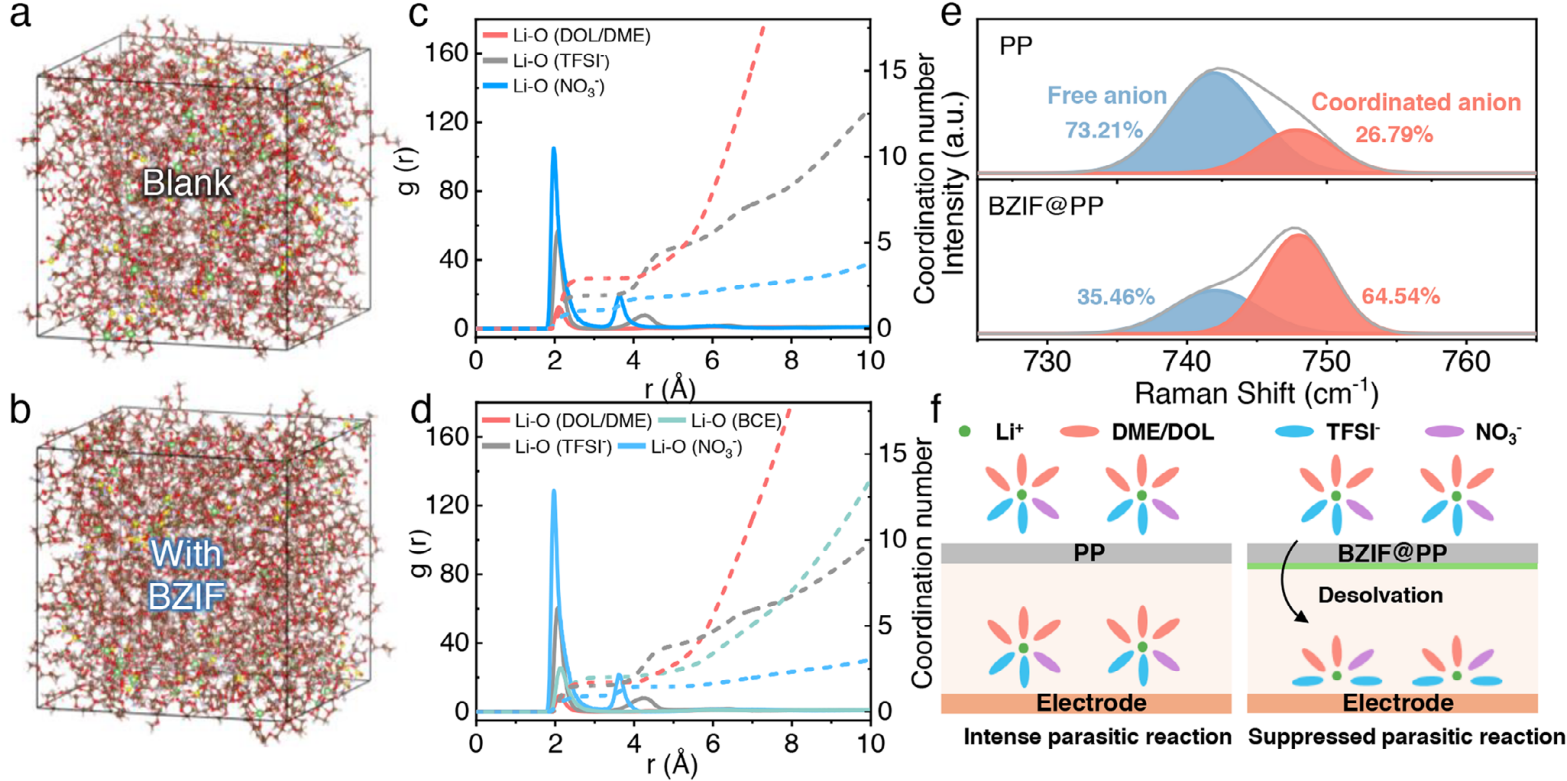
3D snapshots of the MD simulation for the solvation environment (a) in blank electrolyte (1 M LiTFSI in DOL/DME with 2 wt.% LiNO_3_) and (b) in the presence of BZIF interacting with the electrolyte. Radial distribution function (RDF) curves of the Li^+^ solvation structure (c) in blank electrolyte and (d) in the presence of BZIF interacting with the electrolyte. (e) Raman spectra of separators soaked in the electrolyte from the cycled symmetric cells. (f) Schematic diagram of the desolvation mechanism of BZIF ion channels.

It is well known that the composition and structure of the SEI layer largely depend on the Li^+^ solvation structure [45, 46]. To gain a deeper understanding of the formation of the inorganic‐rich SEI layer, molecular dynamics (MD) simulations were employed to investigate the effect of biomimetic ion channels on Li^+^ solvation structure at the anode interface. The 3D snapshots from the MD simulations, along with the corresponding radial distribution function (RDF) results, are presented in Figure 4a–d. For the blank electrolyte (1 M LiTFSI in DOL/DME with 2 wt.% LiNO_3_), DOL/DME serves as the dominant solvent in the first Li^+^ solvation shell (∼2.66 Å), with an average coordination number of 2.95. Additionally, the TFSI^−^ and NO_3_^−^ anions contribute to the Li^+^ coordination structure, with coordination numbers of 1.96 for TFSI^−^ and 1.09 for NO_3_^−^. However, the Li^+^ solvation structure at the interface between the separator and the lithium metal anode is significantly changed when the BZIF bionic ion channels are adopted. Specifically, TFSI^−^ and DOL/DME are the main components in the first Li^+^ solvation shell. Among them, the average Li^+^ coordination number with DOL/DME is dramatically reduced to 1.67, while the Li^+^ coordination number with TFSI^−^ is slightly decreased to 1.53. This indicates that the bionic ion channels promote the Li^+^ desolvation process due to the strong coordination effect between Li^+^ and crown ether sites, which can weaken the coordination capability of DME/DOL solvent molecules with Li^+^, thereby reducing the decomposition of DME/DOL solvent molecules on the lithium anode surface. Raman spectroscopy was employed to further confirm the modulation of the Li^+^ solvation structure. The S‐N‐S stretching peaks of the TFSI^−^ anion can be divided into two peaks corresponding to free anions and coordinated anions, respectively. As shown in Figure 4e, the introduction of the bionic ion channels increases the coordinated anion content from 26.79% to 64.54%. This indicates that more contact ion pairs (CIPs) or aggregates (AGGs) are formed in the Li^+^‐coordinated sheath due to the competitive coordination of crown ether sites [28, 47]. The desolvation mechanism of the BZIF ion channels is illustrated in Figure 4f. For the bare PP separator, a large number of active solvent molecules are exposed on the surface of the lithium anode, leading to intense side reactions and rapid degradation of battery performance. In contrast, when introducing the biomimetic ion channels, the interaction between Li^+^ and crown ether sites promotes the desolvation process of Li^+^, thus suppressing the parasitic reactions caused by active solvent molecules, which is responsible for the thin, compact, and inorganic‐rich SEI.

**FIGURE 5:**
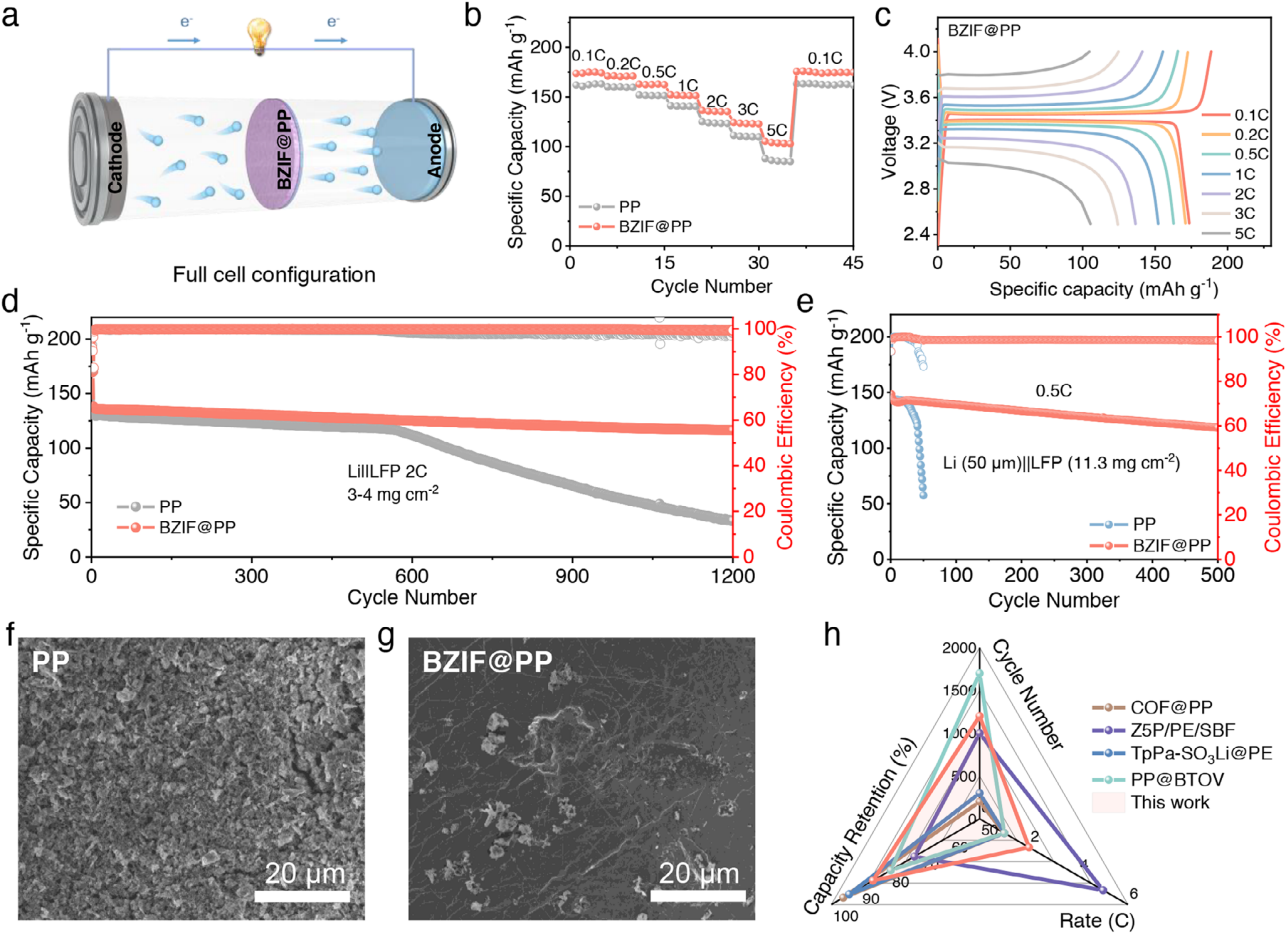
(a) Schematic construction of the Li||BZIF@PP||LFP full cell. (b) Rate performance of Li||LFP cells at the current densities from 0.1 to 5C. (c) The charge and discharge curves of Li||LFP cells at various current densities with the BZIF@PP separator. (d) Long‐term cycling performance of Li||LFP cells at 2C. (e) Cycling performance of thin Li (50 µm)||LFP (11.3 mg cm^−2^) cells at 0.5C with PP and BZIF@PP separators. SEM images (top view) of cycled Li anode from cycled thin Li (50 µm)||LFP (11.3 mg cm^−2^) cells with (f) PP and (g) BZIF@PP separators. (h) Electrochemical performance comparison of full cells with previously reported works.

To further evaluate the practicality of the BZIF ion channels, full cells were assembled by matching Li anode with LiFePO_4_ (LFP) cathode to assess their rate performance and long‐term cycling stability (Figure 5a). As shown in Figure 5b, the discharge specific capacities of Li||BZIF@PP||LFP cell can reach 174.87, 170.91,162.78, 152.16, 136.46, 124.26, and 105.35 mAh g^−1^ at various rates of 0.1, 0.2, 0.5, 1, 2, 3, and 5C, respectively, which are significantly higher than those of the full cells with PP separators (160.76, 159.55, 151.27, 140.42, 123.36, 109.64, and 84.75 mAh g^−1^). The corresponding charge and discharge curves at different current densities indicate that the Li||BZIF@PP||LFP full cells exhibit lower polarization, demonstrating that the BZIF ion channels enhance Li^+^ diffusion and reduce the internal resistance of the cells (Figure 5c and Figure S16).

Further long‐term cycling stability tests, shown in Figure 5d, reveal that full cells with BZIF ion channels retain a higher capacity of 86% at 2C after 1200 cycles, whereas the Li||PP||LFP full cells only retain 25%. The corresponding charge and discharge curves show that the Li||BZIF@PP||LFP full cells exhibit a smaller overpotential compared to the full cells with PP separators (Figure S17), which is also confirmed by the CV results (Figure S18). Postcycling analysis reveals a significantly smoother and denser Li metal anode morphology following 1200 cycles at 2C with BZIF ion channels, compared to that cycled with PP separators (Figure S19). EIS was also employed to assess the internal resistance of the full cells (Figure S20). The results demonstrate that the full cells with BZIF ion channels have lower interfacial resistance both before and after cycling. This suggests that the BZIF ion channels are beneficial to fast Li^+^ transport and promote a more stable electrode/electrolyte interface, thereby achieving improved rate and cycling performance. Moreover, when paired with high‐mass‐loading LFP cathodes (11.3 mg  cm^−2^, ∼1.7 mAh cm^−2^) and a thin Li anode (50 µm), the Li||BZIF@PP||LFP full cells still maintain superior cycling stability (82% capacity retention at 0.5C after 500 cycles) and rate performance (Figure 5e and Figure S21). Post‐cycling analysis of the Li anodes after 500 cycles reveals a porous, mossy structure with substantial dead Li when the PP separator is used (Figure 5f). In contrast, the anode from the BZIF@PP‐based cell exhibits a denser morphology with significantly less dead Li (Figure 5g), corroborating the superior interfacial stability that enables long‐term cycling. To prove the applicability of BZIF ion channels in high‐voltage systems, Li||LiNi_0.8_Co_0.1_Mn_0.1_O_2_ (NCM811) full cells were assembled. As shown in Figure S22, cells with BZIF bionic ion channels show enhanced cycling stability and rate performance. Furthermore, when compared to previously reported studies on separator modification (Figure 5h) [28, 48, 49, 50], the full cells with the BZIF ion channels show a moderate electrochemical performance.

## 3 Conclusions

In summary, we engineered a bioinspired ion‐channel structure, consisting of BCE encapsulated within a ZIF‐7 matrix (BZIF), as a functional separator coating to stabilize the anode/electrolyte interface in lithium metal batteries. This design mimics biological ion channels, creating fast Li^+^ conduction pathways that establish a Li^+^‐enriched interfacial zone. The bionic ion channels homogenize Li^+^ flux and reduce concentration polarization between the anode and the bulk electrolyte, enabling dendrite‐free lithium deposition. Furthermore, the crown ether sites of bionic ion channels weaken Li^+^‐solvent coordination via ion‐dipole interactions and facilitate the desolvation of Li^+^, enriching the solvation sheath with anions. Subsequent decomposition of this anion‐rich environment generates an inorganic‐rich SEI (LiF/Li_3_N) with high Li^+^ conductivity, enhancing anode interfacial stability. As a result, Li||Li symmetric cells with BZIF ion channels exhibit excellent long‐term stability over 1500 h without short‐circuit. The assembled Li||BZIF@PP||LFP full cells also show an inspiring capacity retention of 86.0% at 2C after 1200 cycles with an average CE above 99.8%. Finally, this work demonstrates a synergistic strategy for stabilizing lithium metal anodes through molecular‐level regulation of Li^+^ flux and interfacial chemistry.

## Conflicts of Interest

The authors declare no conflicts of interest.

## Supporting information




**Supporting File**: smll74056‐sup‐0001‐SuppMat.docx.

## Data Availability

The data that support the findings of this study are available from the corresponding author upon reasonable request.
