# Supplementary material for: Biomimetic Ion Channel Design for Simultaneous Lithium‐Ion Flux Regulation and Interfacial Stabilization in Lithium Metal Batteries
Source: Small. 2026 Jun 3;22(42):e74056. doi: 10.1002/smll.74056 (PMC13410453; doi:10.1002/smll.74056)
Supplement: Supplementary file 1 — Supporting File: smll74056‐sup‐0001‐SuppMat.docx. [file SMLL-22-e74056-s001.docx]

**Supporting Information**

Biomimetic Ion Channel Design for Simultaneous Lithium-Ion Flux Regulation and Interfacial Stabilization in Lithium Metal Batteries

*Qian Cheng^1,2,3#^, Ke Fan^4#^, Jun-Ming Cao^1^, Zezhou Lin^1^, Jiamin Fu^5^, Shengjie Xia^2,3^, Chao Wang^2,3^, Yao Liu^1,2,3^, Simeng Zhang^2,3^, Changhong Wang^2,3^*, Xueliang Sun^2,3^*, and Haitao Huang^1,6^**

^1^ Q. Cheng, J. Cao, Z. Lin, Y. Liu, Prof. H. Huang

Department of Applied Physics, The Hong Kong Polytechnic University, Hong Kong 999077, China

E-mail: aphhuang@polyu.edu.hk

^2^ Q. Cheng, S. Xia, C. Wang, Y. Liu, S. Zhang, Prof. C. Wang, Prof. X. Sun

Eastern Institute for Advanced Study, Ningbo Institute of Digital Twin, Eastern Institute of Technology, Ningbo 315200, China

E-mail: cwang@eitech.edu.cn; xsun@eitech.edu.cn

^3^ Q. Cheng, S. Xia, C. Wang, Y. Liu, S. Zhang, Prof. C. Wang, Prof. X. Sun

Zhejiang Key Laboratory of All-Solid-State Battery, Ningbo Key Laboratory of All-Solid-State Battery, Ningbo 315200, China

^4^ K. Fan

School of Materials Science and Engineering, Anhui University, Hefei 230601, China

^5^ J. Fu

Department of Mechanical and Materials Engineering, University of Western Ontario, London, Ontario, N6A 5B9, Canada

^6^ Prof. H. Huang

Research Institute for Advanced Manufacturing, The Hong Kong Polytechnic University, Hong Kong 999077, China

**Experimental Section**

**Synthesis of BCE@ZIF-7 (BZIF)**

The BZIF particles were synthesized via a one-step encapsulation method. In a typical preparation process, 1.90 g benzimidazole was first dissolved in 20 mL DMF. Then 0.60 g zinc nitrate hexahydrate (Zn(NO_3_)_2_⋅6H_2_O) and 0.12g benzo-12-crown-4-ether (BCE) were dissolved in 20 mL DMF. The latter solution was slowly added into the former under stirring. The solution was then aged for 6 h after stirring for 30 min at room temperature. Subsequently, the white precipitates were collected by centrifugation at 8500 rpm for 10 min and washed with methanol three times. Finally, the product was dried at 60 °C under vacuum overnight. The synthesis of ZIF-7 was similar except that BCE was not added.

**Preparation of the BZIF@PP separator**

The slurry was prepared by mixing the BZIF powder and poly vinylidene fluoride (PVDF) binder with the mass ratio of 9:1 in N-methyl-2-pyrrolidinone (NMP), which was then coated onto polypropylene (PP) separator. After drying at 80 °C for 12 h, the modified separators were cut into discs with a diameter of 19 mm.

**Electrochemical measurements**

The cathode slurry, consisting of 80% LiFePO_4_ powders, 10% PVDF binder, and 10% Super P in NMP, was coated onto aluminum foil. The cathodes were then dried in a vacuum oven at 80 °C for 12 h. The average mass loading of electrodes was about 3-4 mg cm^-2^ with a diameter of 12 mm. The CR-2032 type coin cells were assembled in an argon-filled glove box. The electrolyte was 1.0 M lithium bis(trifluoromethane sulfonyl) imide (LiTFSI) in a mixed solvent of 1,3-dioxolane (DOL) and 1,2-dimethoxyethane (DME) (1:1 in volume) with 2% LiNO_3_. For the NCM811 cathode with a mass loading of 5-6 mg cm^-2^, the slurry was obtained by mixing NCM811 powders, Super P, and PVDF binder with a weight ratio of 8:1:1 in NMP solvent. The corresponding electrolyte is composed of 1.0 M LiPF_6_ in EC/DEC (1:1 by vol%).

Electrochemical Impedance Spectroscopy (EIS), cyclic voltammetry (CV), and Tafel polarization tests were performed using a Solartron electrochemical workstation. EIS measurements were conducted with an amplitude of 10 mV across a frequency range from 1 MHz to 0.1 Hz. CV measurements of Li||Cu half cells were performed over a voltage range of 0-2.5 V with a fixed sweep rate of 1 mV s^-1^. Tafel tests were conducted at a scan rate of 0.5 mV s^-1^ over a voltage range of -0.2 V to 0.2 V. The Li-ion transference number (t_Li_^+^) was determined by direct current (DC) polarization of a Li||Li symmetric cell under a DC voltage of 10 mV. Galvanostatic charge-discharge tests of Li||Cu cells and Li||Li symmetric cells were carried out by a LAND CT2001A battery system. The LFP||Li full cells were cycled from 2.5 to 4.0 V with initial three activation cycles at 0.1 C. The NCM811||Li full cells were cycled from 3.0 to 4.3 V with initial three activation cycles at 0.1 C. All electrochemical tests were conducted at room temperature (25 °C).

**Characterizations**

The X-ray diffraction (XRD) data were obtained by a Bruker D8 Advanced X-ray diffractometer (Cu Kα, λ = 1.5418 Å). The scanning electron microscopy (SEM, Hitachi S-4800) was employed to observe the morphologies of BZIF particles, modified separator and lithium deposition. The pore size distribution of samples were evaluated by the Brunauer-Emmett-Teller method (BET Micromeritics ASAP2460). Fourier transform infrared (FTIR) spectra were recorded using a Nicolet iS20 spectrometer in attenuated total reflection (ATR) mode. Raman spectroscopy (DXR3, 532 nm filter) was performed to investigate the properties of pure PP and BZIF@PP separators soaked in the electrolytes. X-ray photoelectron spectroscopy (Thermo SCIENTIFIC ESCALAB 250Xi) was performed to characterize the chemical composition of the SEI layer. Time-of-flight secondary ion mass spectrometry (TOF-SIMS) analyses were carried out on a PHI nanoTOF II (Ulvac-Phi) instrument in high mass resolution mode, utilizing a 30 keV Bi^3+^ ion beam for the acquisition phase and a 3 kV Ar^+^ ion beam for the sputter phase. The typical areas analyzed and sputtered were 100 × 100 μm^2^. For XPS and TOF-SIMS measurements, Li electrodes were extracted from Li||Li symmetric cells after 10 cycles at 1 mA cm^-2^ with a cutoff capacity of 1 mAh cm^-2^. The electrodes were then transferred to the instrumental chambers from an Ar-filled glovebox without any exposure to air.

**Finite element simulation**

The Li^+^ concentration distribution was simulated using the finite element method under adaptive meshing on COMSOL Multiphysics 6.1 platform. The behavior of Li^+^ was determined by diffusion and electric field migration based on Fick's law, the Nernst-Planck equations, and the condition of electrical neutrality based on Li ions and anions. The bottom region of the model was used as the electrode to reduce the computational cost, and the size of the electrolyte solution was set to 30 × 30 μm^2^, with BZIF layer (~5 μm) placed on the lithium metal surface. These simplifications can offer a fundamental understanding of an idealized system, although they may not fully reflect real-world conditions. The gridding was done using a free-quadrilateral grid with a maximum grid cell of 0.2 μm and a minimum grid cell of 0.005 μm to improve the accuracy of the computation. The Li^+^ diffusion coefficient in bulk electrolyte was set to 5.7×10^-11^ m^2^ s^-1^, representing a t_Li_^+^ of 0.38 in Fig. S6. In the BZIF functional layer, the Li^+^ diffusion coefficient was set to 2.3×10^-10^ m^2^ s^-1^ based on the data of ionic conductivity and Li^+^ transference number. The model adopted Delicacy boundary conditions. The upper boundary was set to Li^+^ concentration of 1 M and a potential of 0.1 V, and the lower boundary was set to Li^+^ concentration of 0 and a potential with 0 V. The other boundaries are natural boundaries with zero flux.

**Molecular dynamics (MD) simulation**

The classical molecular dynamics (MD) simulations were implemented in the LAMMPS package with the generalized Amber force field (GAFF). The parameters of anions and solvents were adopted from established force field databases. For the LAMMPS simulations, Packmol and Moltemplate codes were constructed to obtain the initial geometric structures and parameters. To simulate the blank electrolyte (1 M LiTFSI in DOL/DME with 2 wt% LiNO_3_), the constructed model contained 100 LiTFSI, 36 LiNO_3_, 715 DOL and 481 DME. To explore the role of BZIF, 193 BCE (rough estimate based on load) were added to the above system. During the MD calculations, the energy minimization was performed with an energy tolerance of 10^-4^ and 1 fs of the time step was set. Next, each system was equilibrated for 7 ns in the NPT ensemble (Nosé-Hoover thermostat/barostat), with the pressure of 1 atm and temperature of 300 K were considered. Another 5 ns simulations were subsequently performed in the NVT ensemble (Berendsen thermostat, 300 K), and the final 4 ns trajectory was then utilized to gather the necessary data for analysis.

**
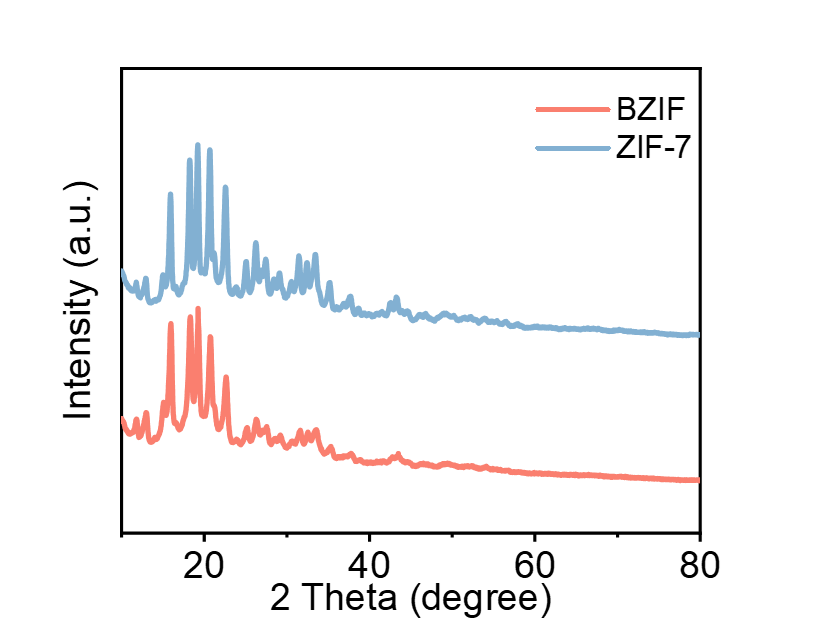
**

**Figure S1.** XRD patterns of ZIF-7 and BZIF.

**
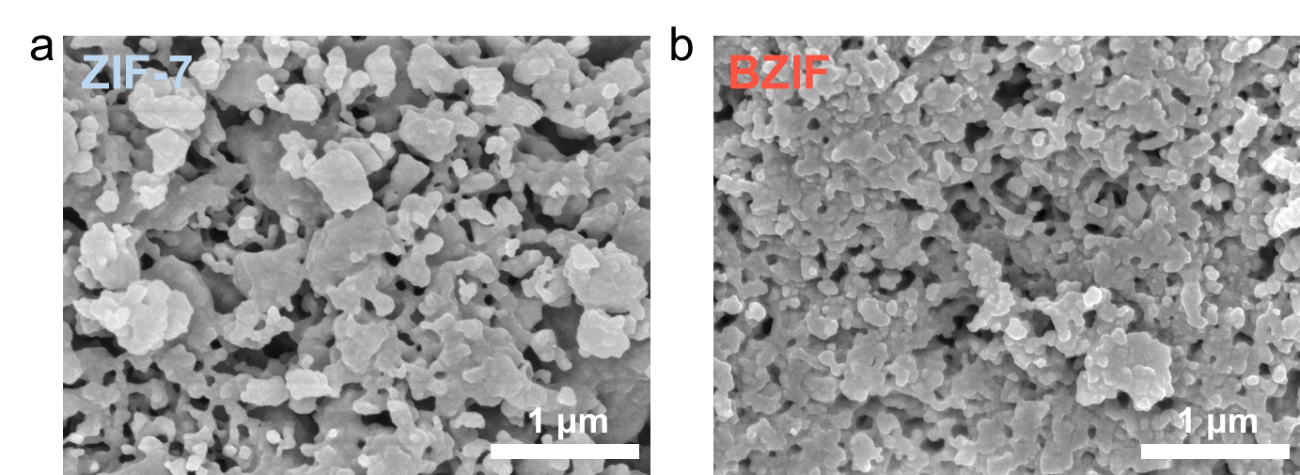
**

**Figure S2.** SEM images of (a) ZIF-7 and (b) BZIF nanoparticles.

**
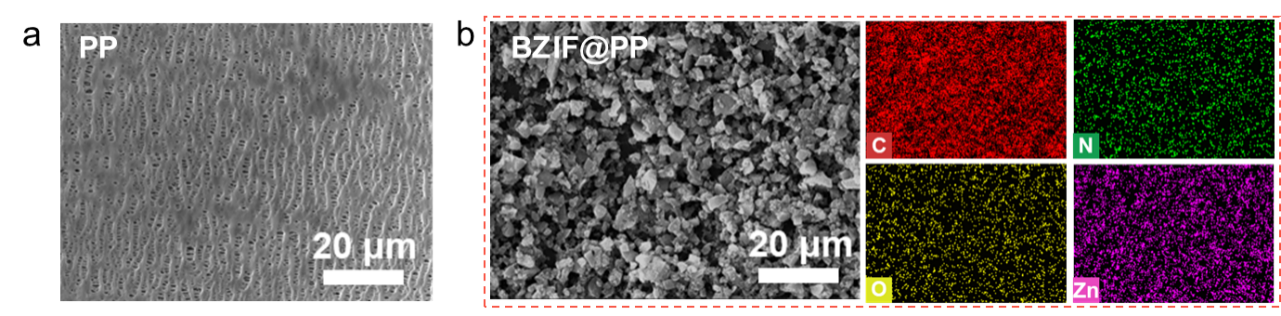
**

**Figure S3.** (a) SEM images of bare PP separator. (b) SEM characterizations of the BZIF@PP separator and the corresponding elemental mapping images.

**
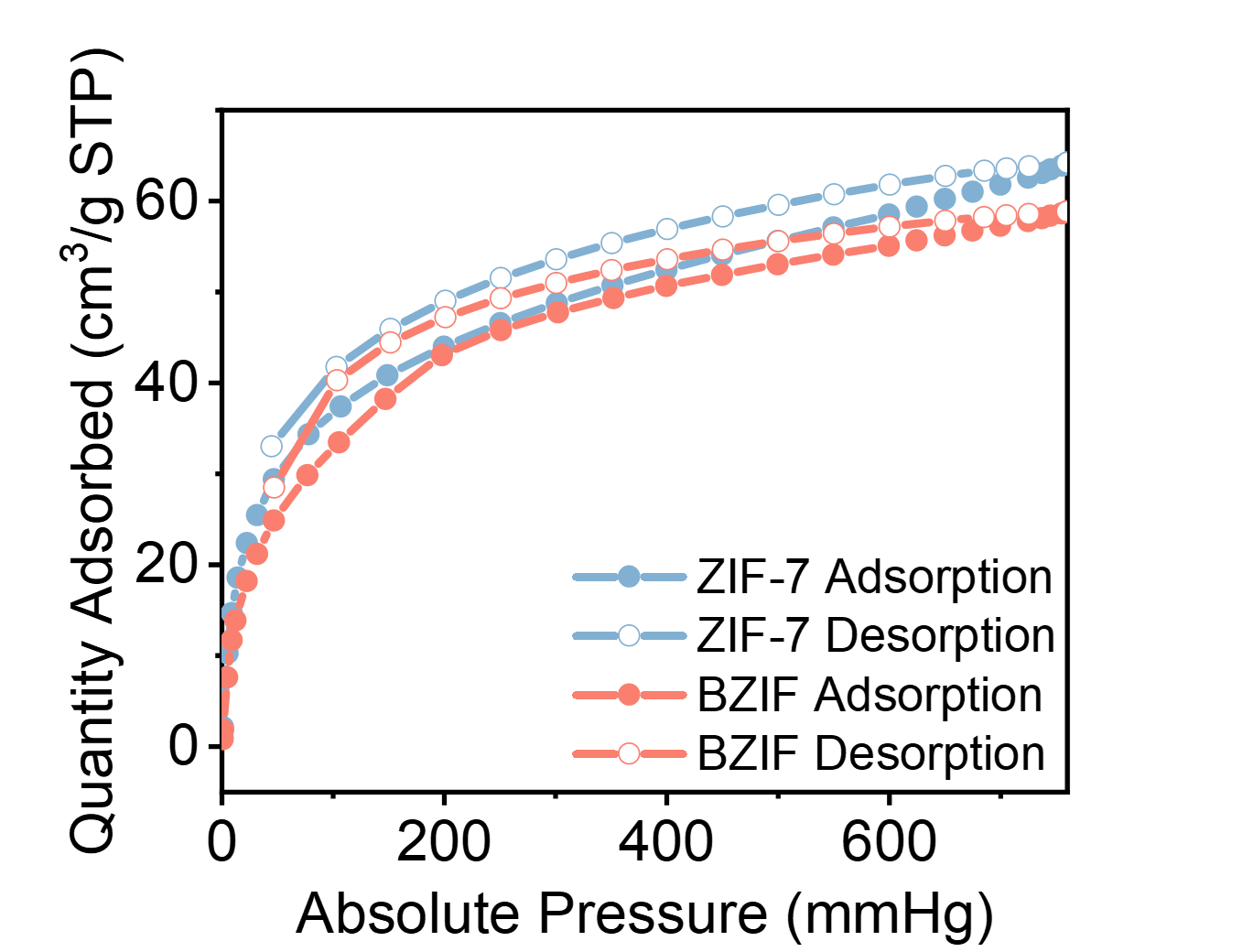
**

**Figure S4.** CO_2_ adsorption/desorption isotherms of ZIF-7 and BZIF particles at 273 K.

**
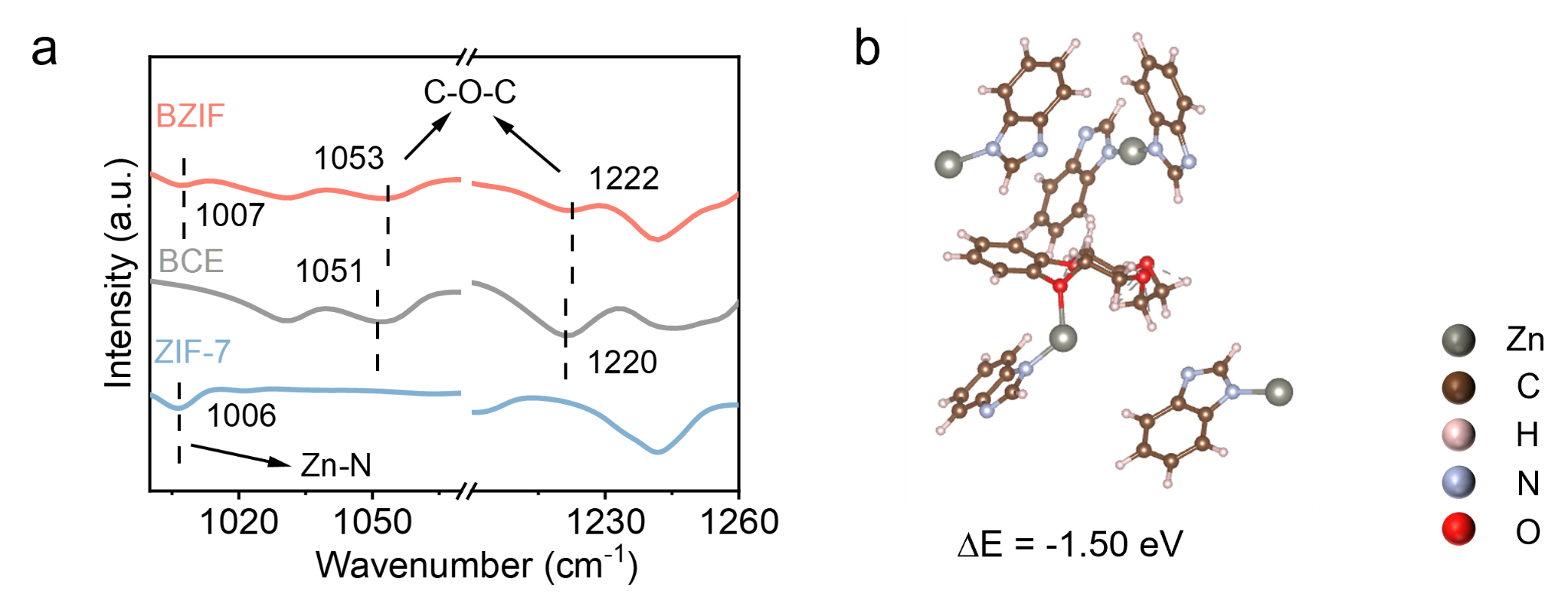
**

**Figure S5.** (a) FT-IR spectra of ZIF-7, BCE and BZIF samples. (b) The binding energy between ZIF-7 and BCE.

**
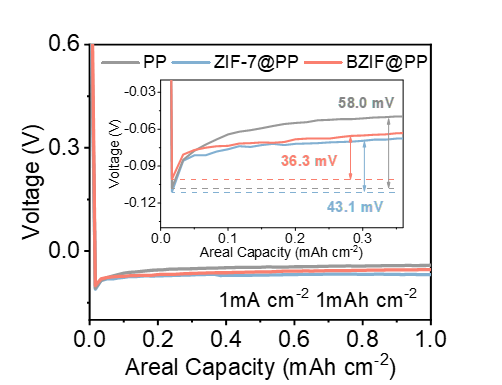
**

**Figure S6.** Voltage-capacity curves (inset: corresponding nucleation overpotentials of Li||Cu half cells with different separators at 1 mA cm^-2^ for 1 mAh cm^-2^).

**
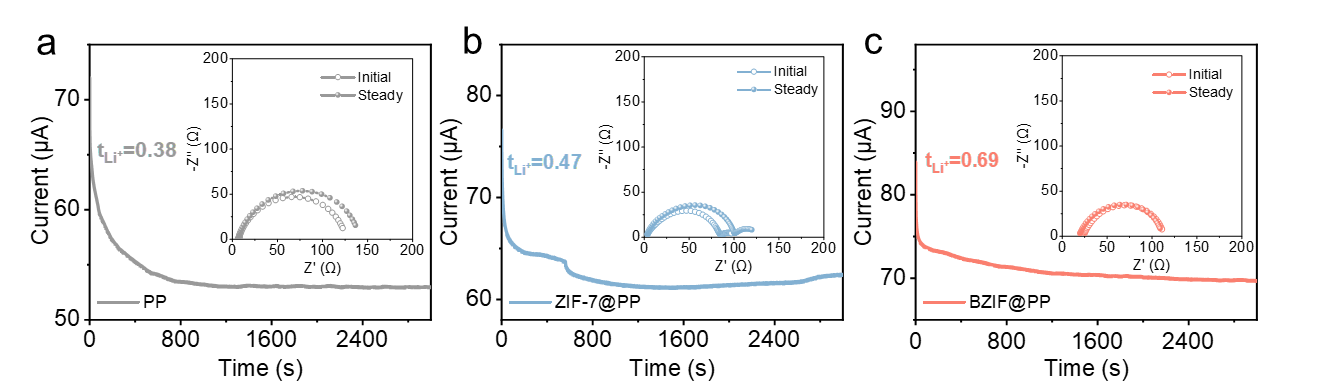
**

**Figure S7.** Chronoamperometry profiles of Li||Li symmetric cells with different separators (inset: Nyquist plots before and after polarization).

**
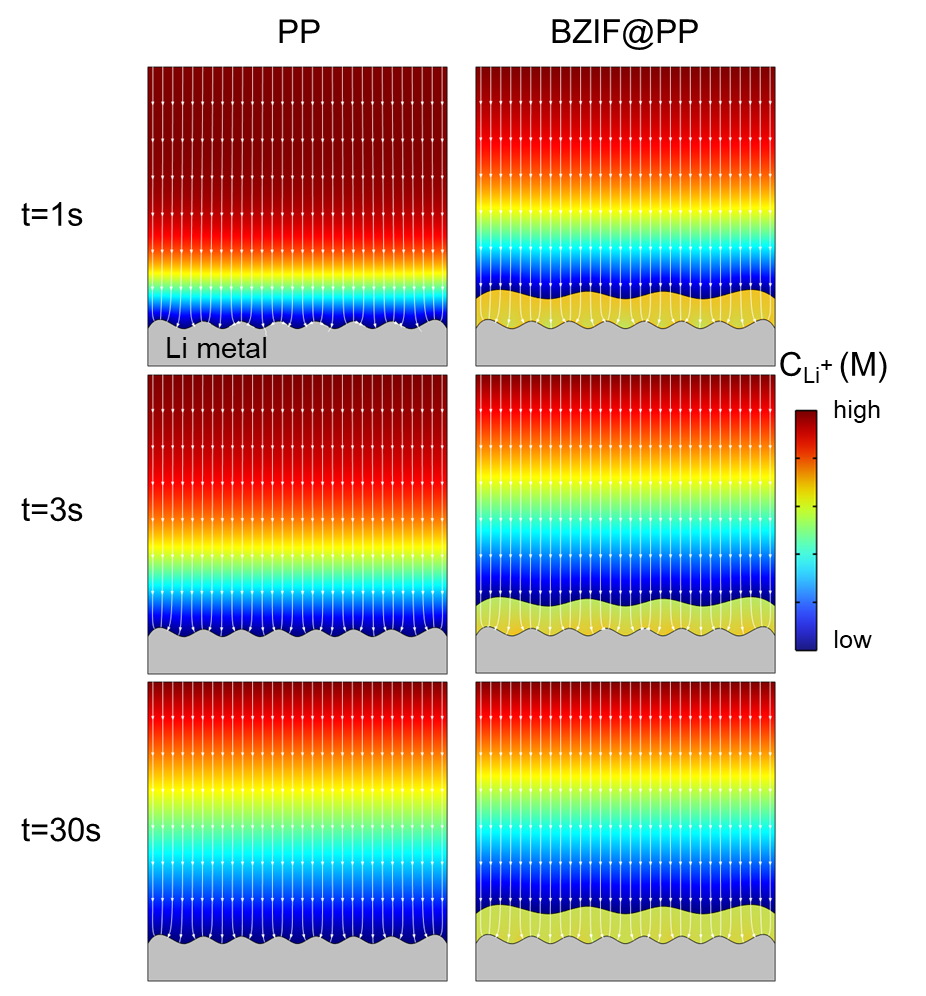
**

**Figure S8.** The simulated Li^+^ concentration distribution in the electrolyte with pure PP, and BZIF@PP separators after 1, 3, and 30 s, respectively.

**
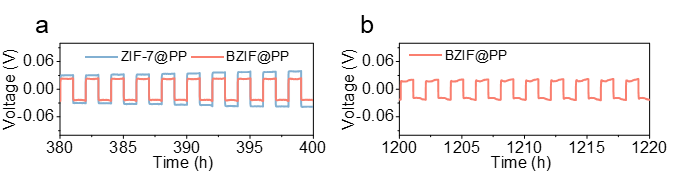
**

**Figure S9.** The corresponding enlarged Li plating/stripping curves for long-term cycling of the Li||Li symmetric cells in Fig. 3c (1 mA cm^-2^, 1 mAh cm^-2^).

**
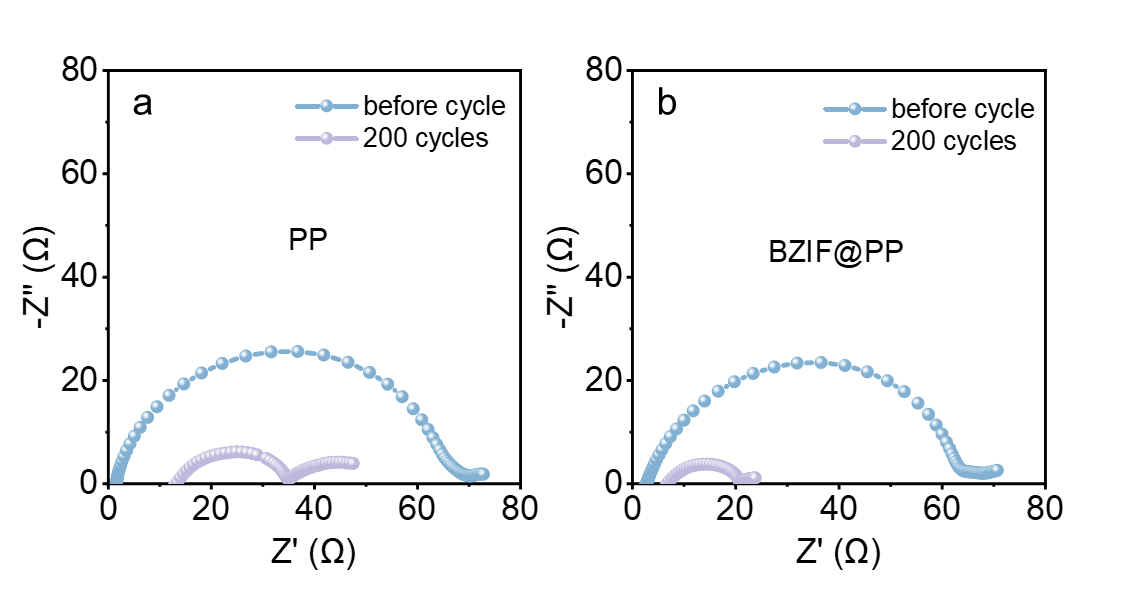
**

**Figure S10.** The Nyquist plots of Li||Li symmetric cells before cycling and after 200 cycles with (a) bare PP separators and (b) BZIF@PP separators.

**
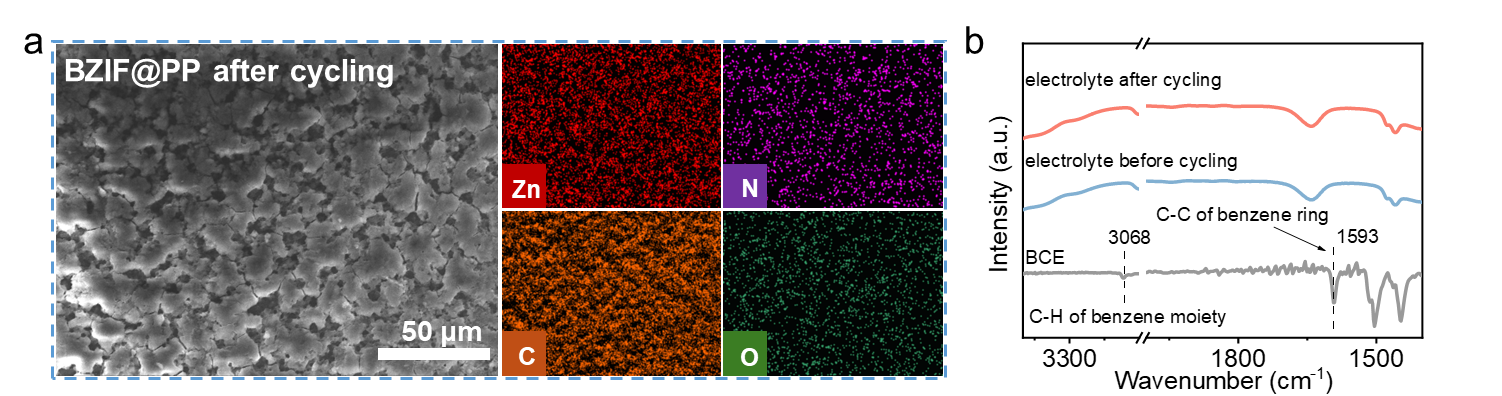
**

**Figure S11.** (a) SEM characterizations of BZIF@PP after cycling and the corresponding elemental mapping images. (b) FT-IR spectra of BCE molecules and the electrolyte before and after cycling with BZIF@PP.

**
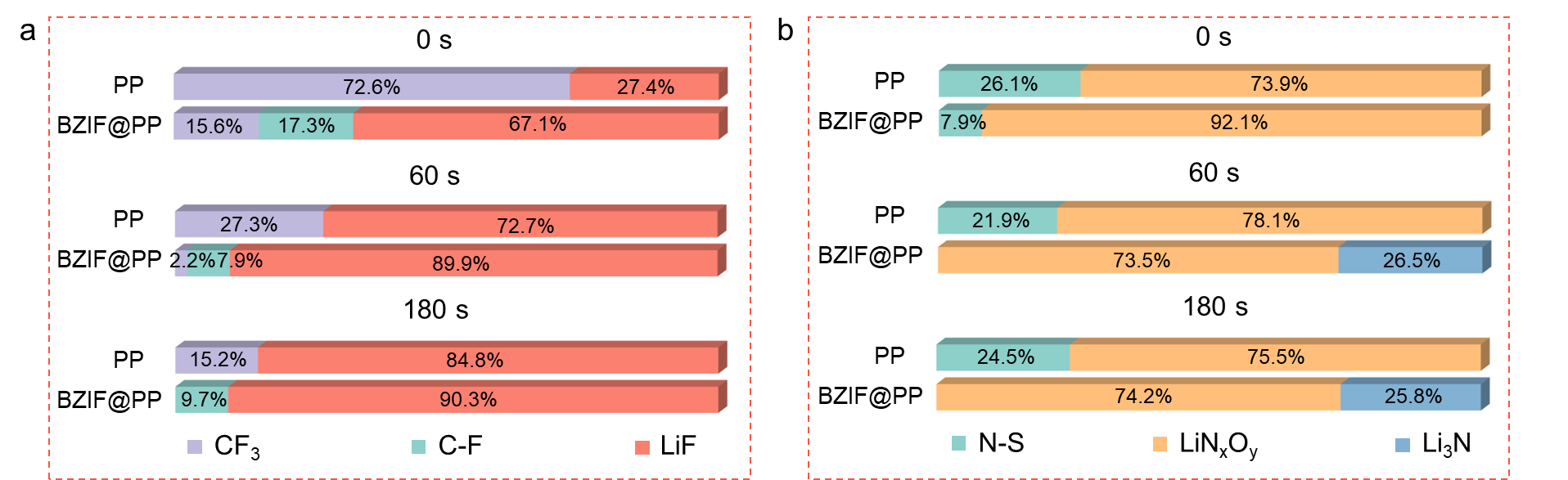
**

**Figure S12.** The content of (a) fluorine-containing species and (b) nitrogen-containing species at different XPS etching depths for the pure PP and BZIF@PP separators.

**
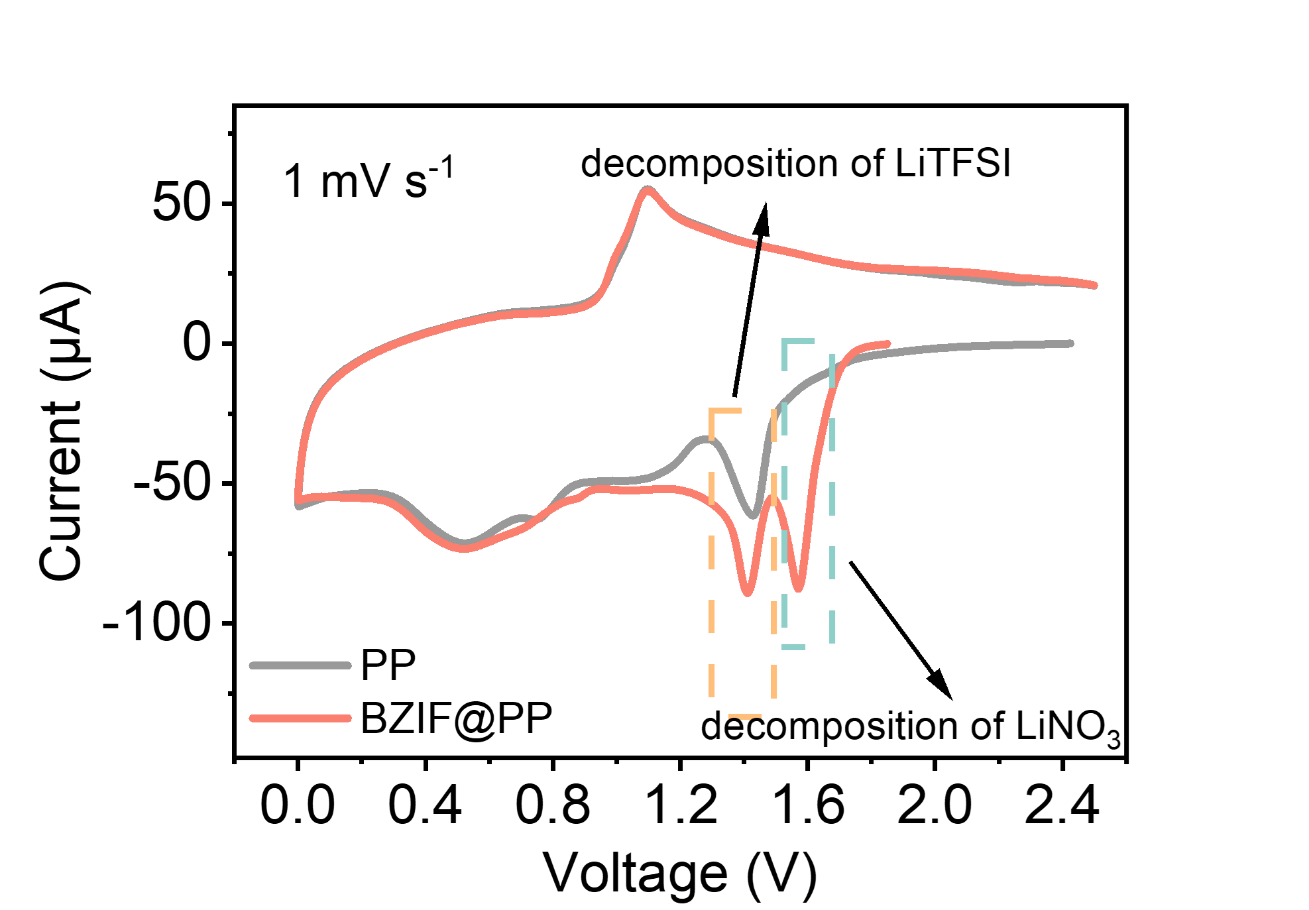
**

**Figure S13.** The cyclic voltammetry (CV) curves of Li||Cu half cells with PP and BZIF@PP separators.

**
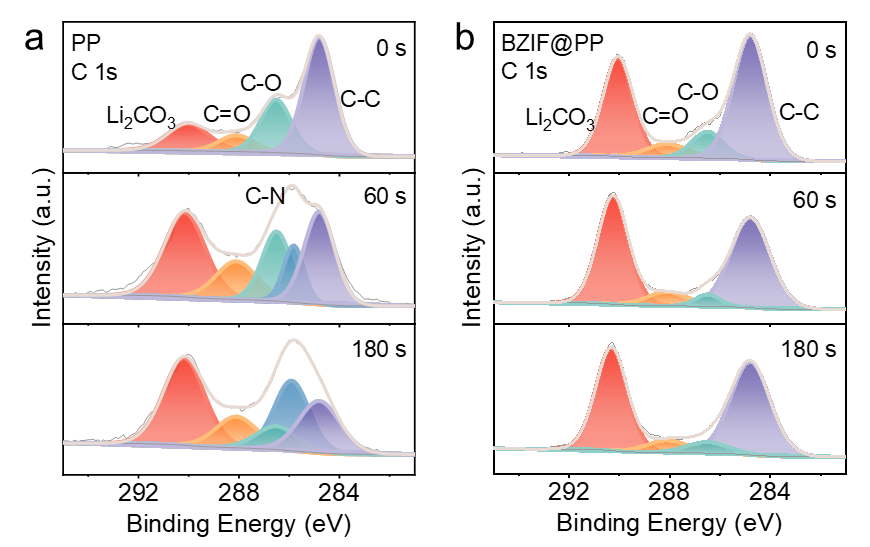
**

**Figure S14.** The XPS depth profiles of C 1s with (a) bare PP separators and (b) BZIF@PP separators for cycled Li anode.

**
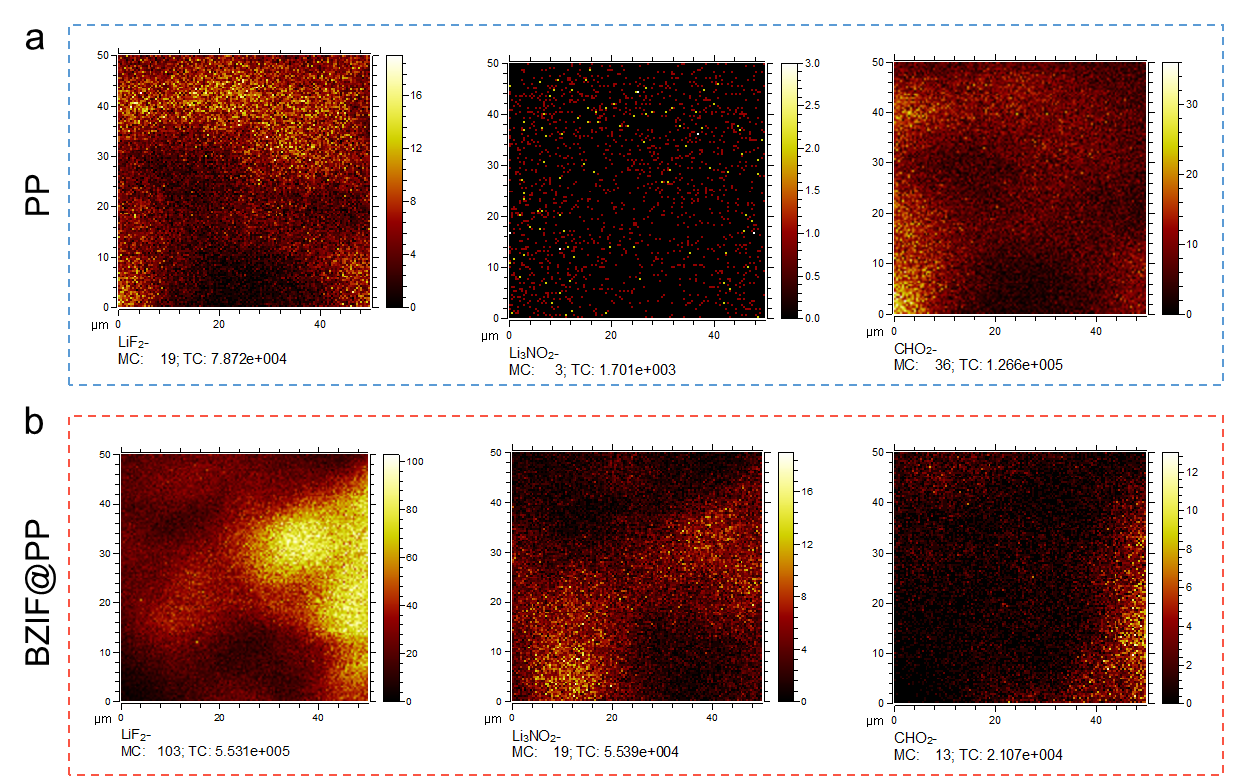
**

**Figure S15.** TOF-SIMS 2D spectra of LiF_2_^-^, Li_3_NO_2_^-^, and CHO_2_^-^ fragments for cycled Li anode with (a) bare PP separators and (b) BZIF@PP separators.

**
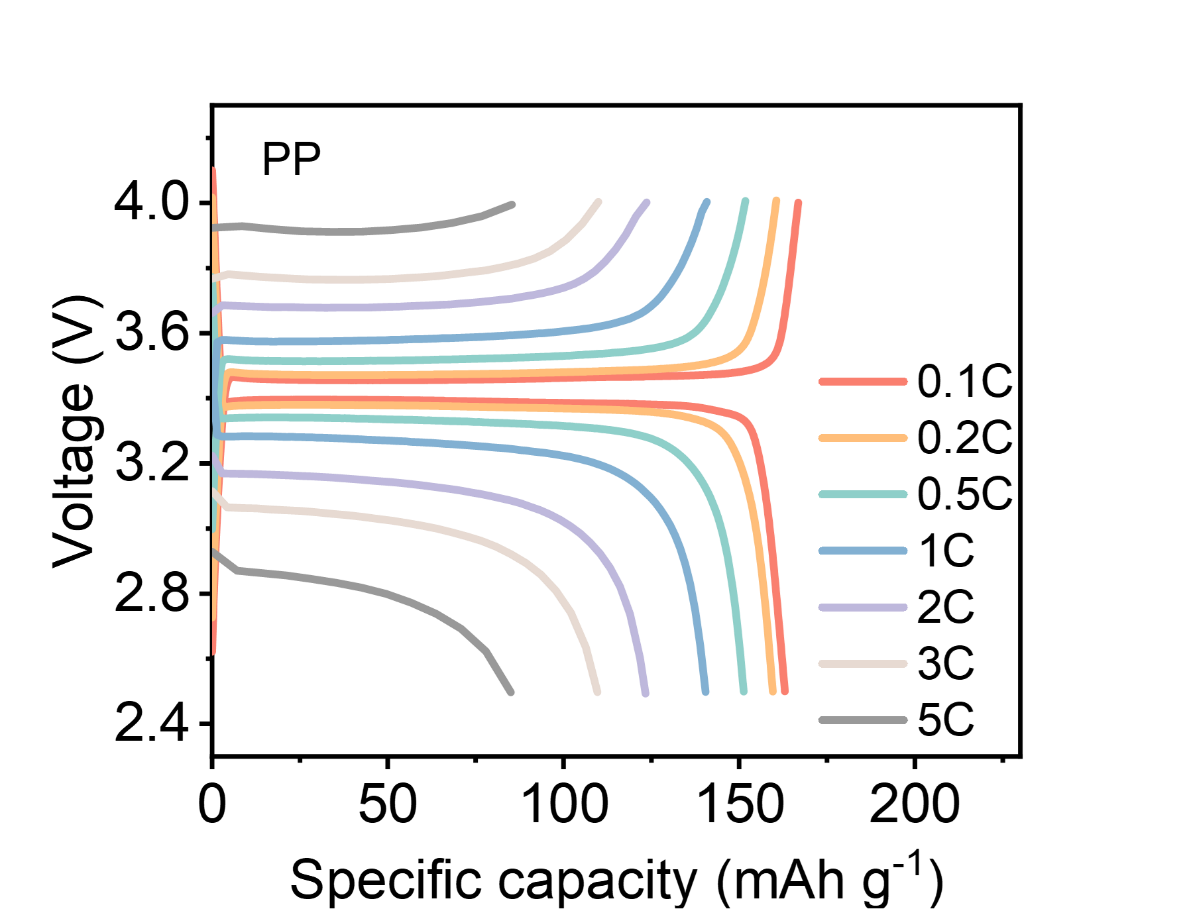
**

**Figure S16.** The charge and discharge curves of Li||LFP cells at various rates with pure PP separators.

**
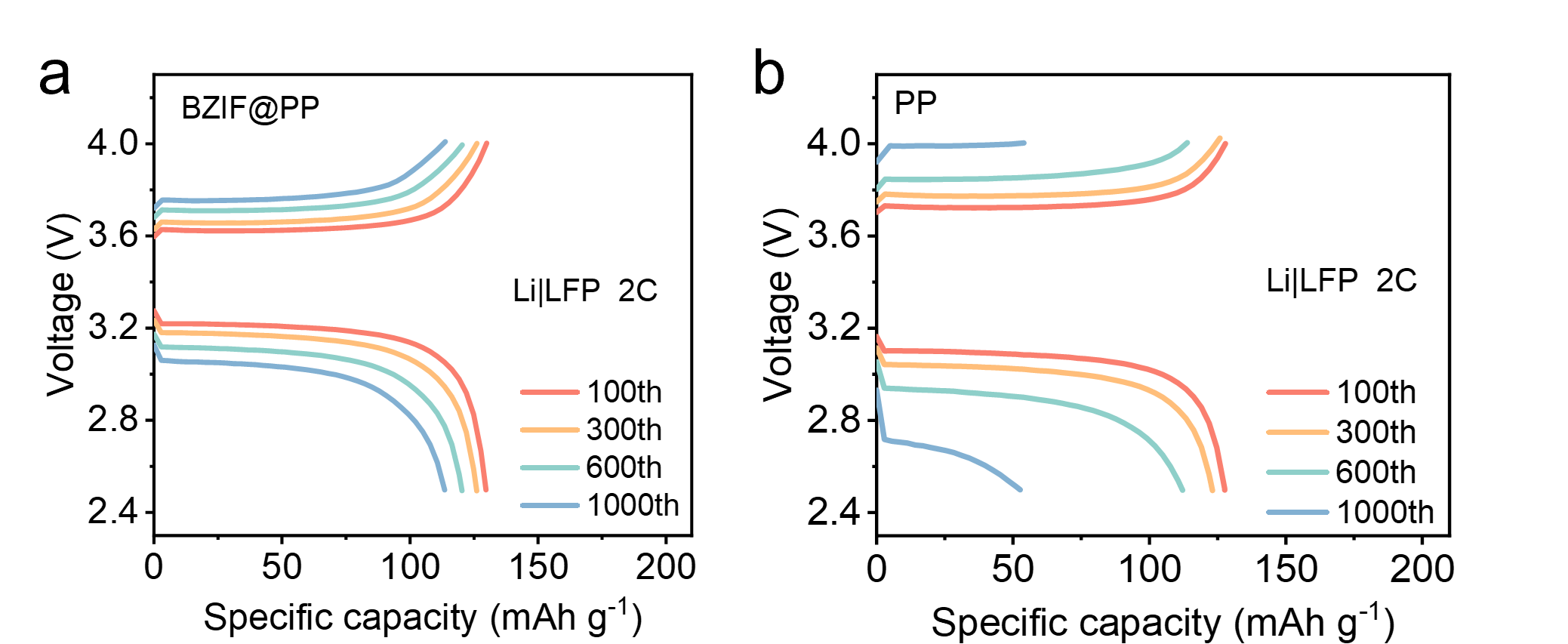
**

**Figure S17.** The charge and discharge curves of Li||LFP full cells at 2 C with (a) BZIF@PP and (b) pure PP separators.

**
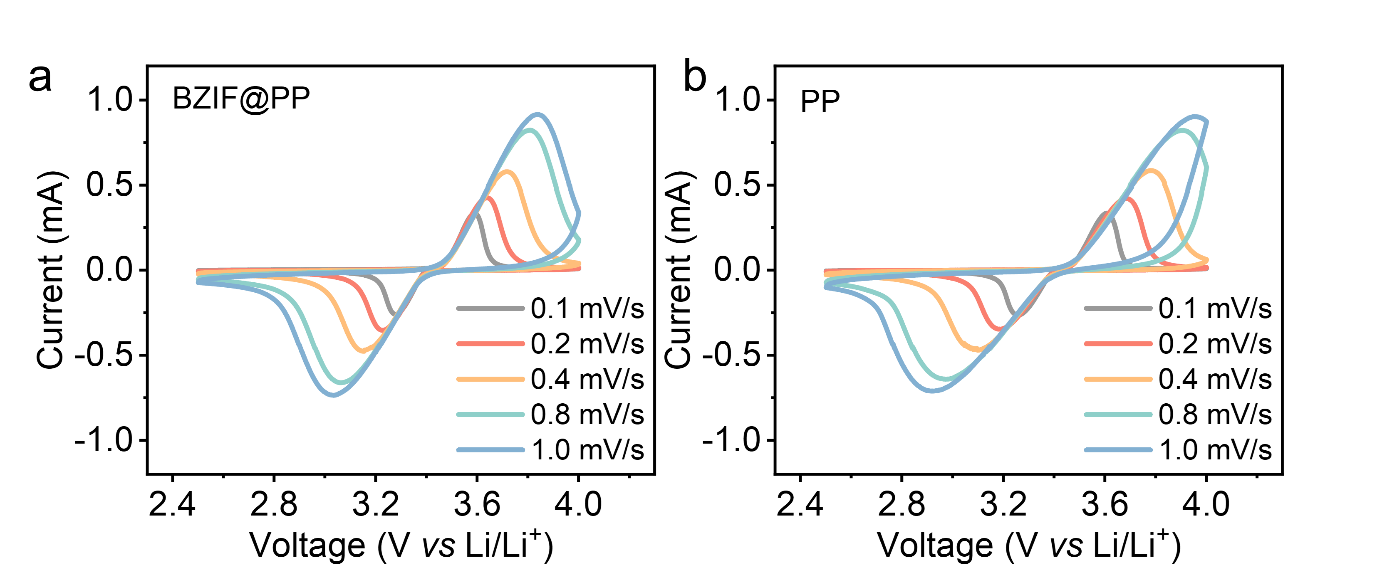
**

**Figure S18.** Cyclic voltammetry (CV) curves of Li||LFP full cells with (a) BZIF@PP and (b) PP separators.

**
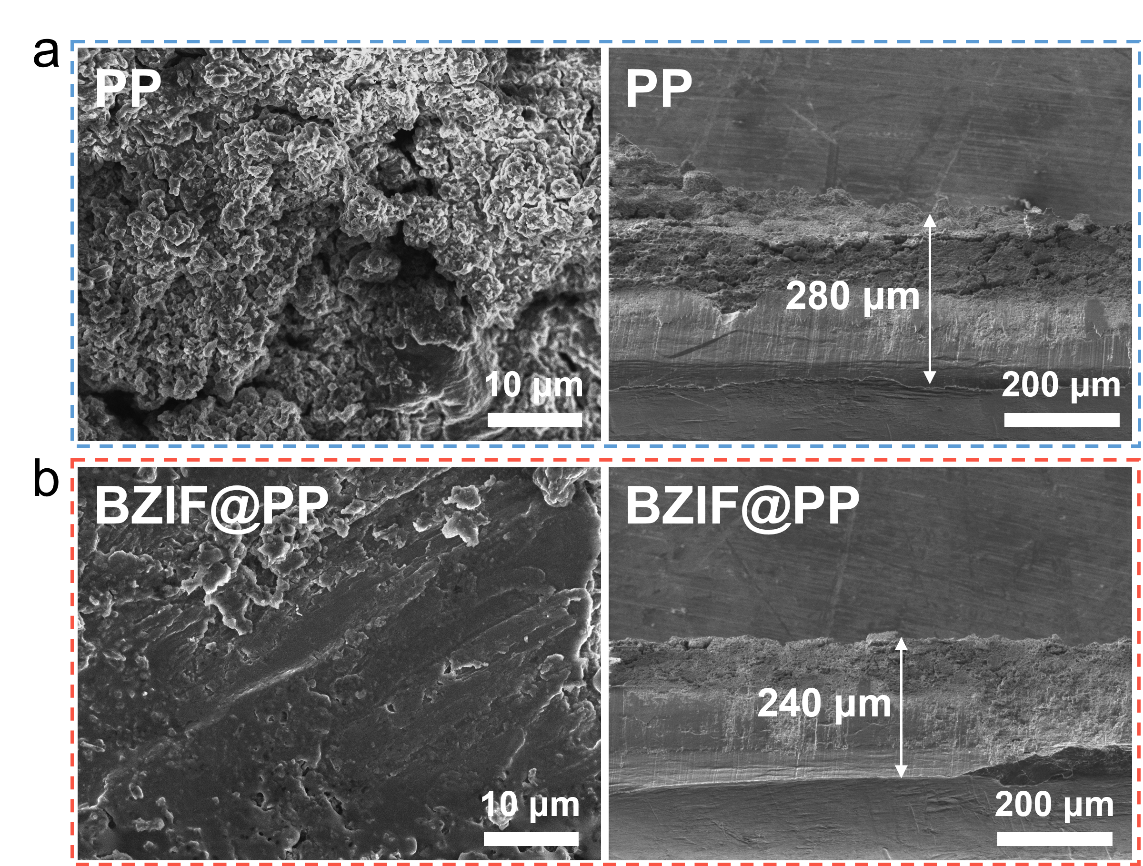
**

**Figure S19.** SEM images of cycled Li metal in top view and cross-section view with (a) bare PP separators and (b) BZIF@PP separators after 1200 cycles at 2C.

**
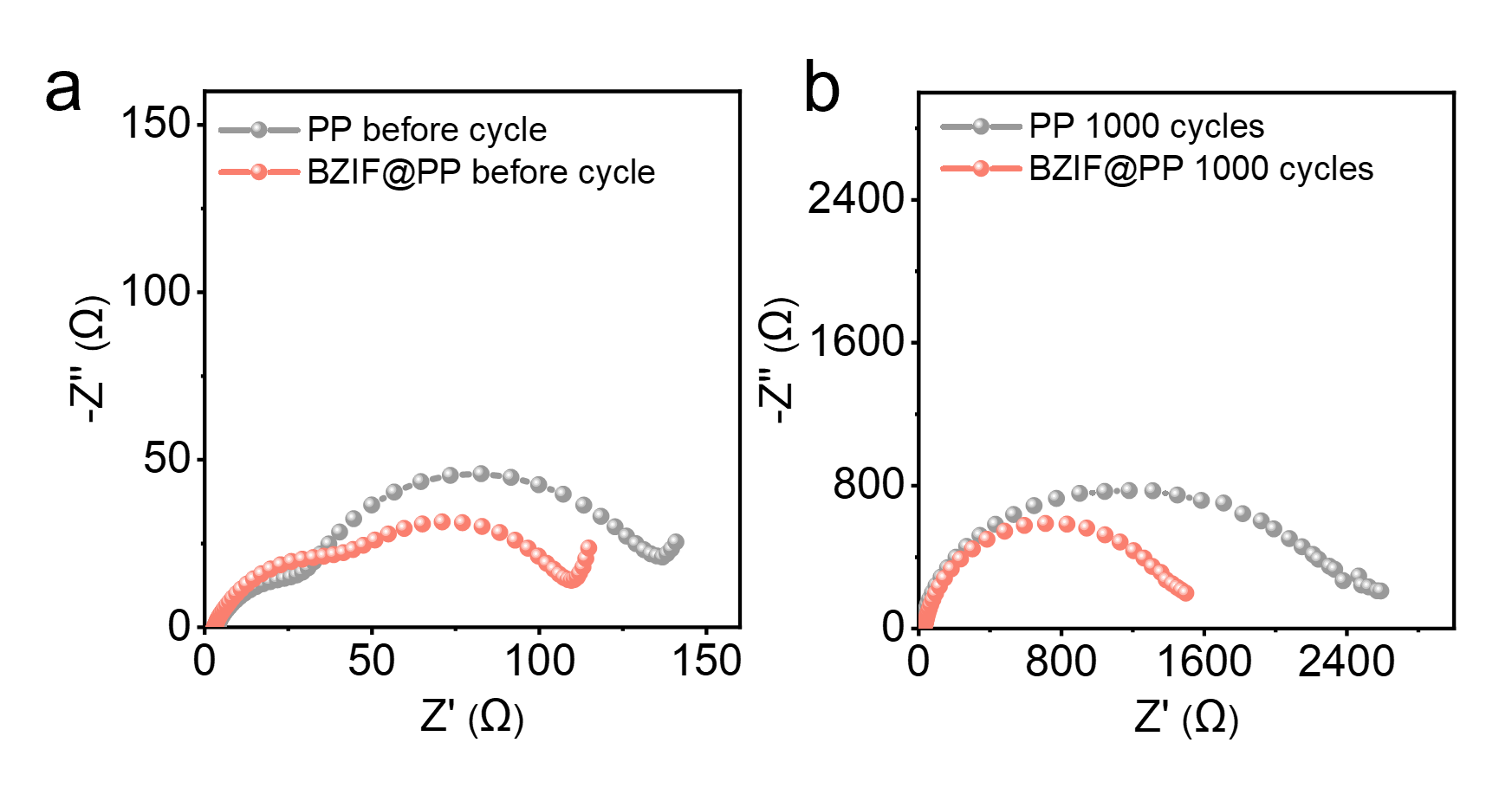
**

**Figure S20.** The Nyquist plots of Li||LFP cells (a) before cycling and (b) after 1000 cycles with bare PP separators and BZIF@PP separators.

**
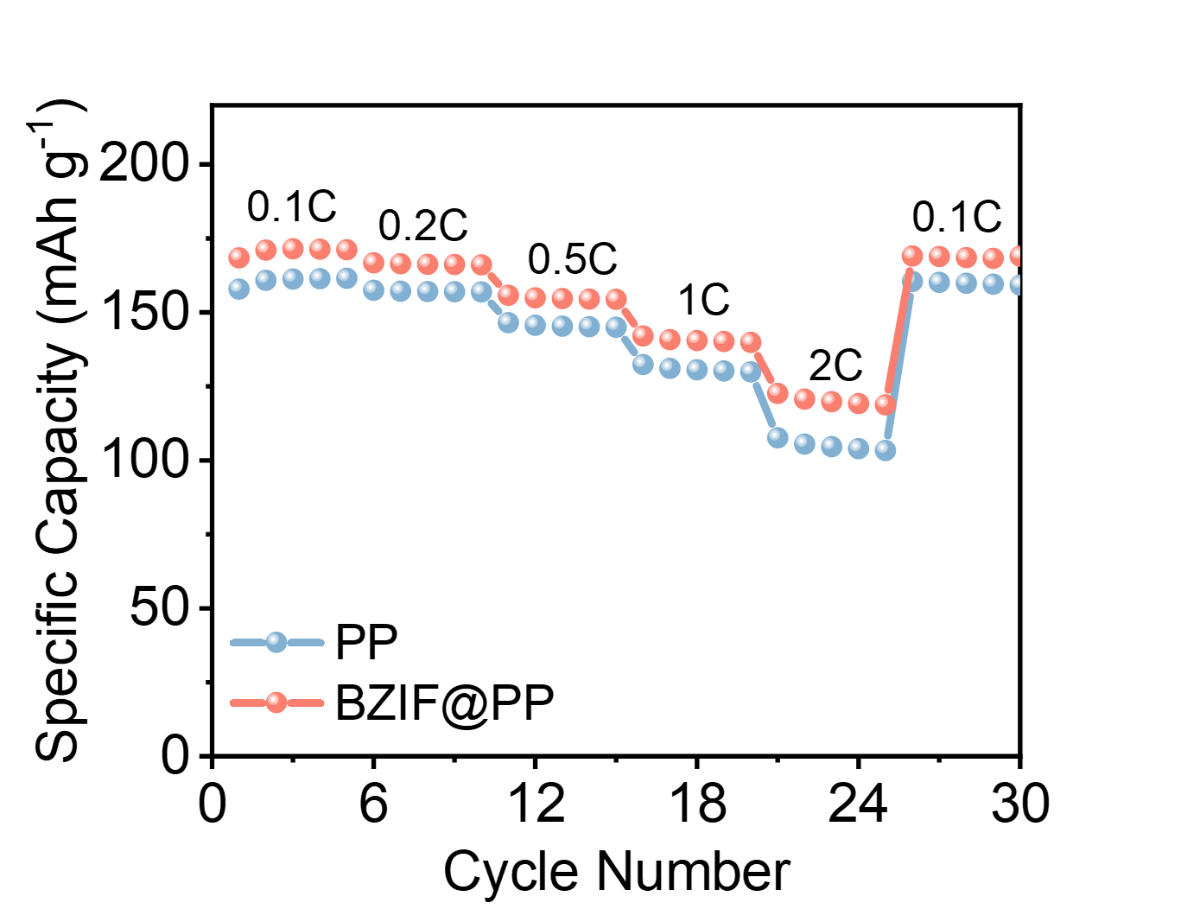
**

**Figure S21.** The rate performance of thin Li (50 μm)||LFP (11.3 mg cm^-2^) cells with PP and BZIF@PP separators.

**
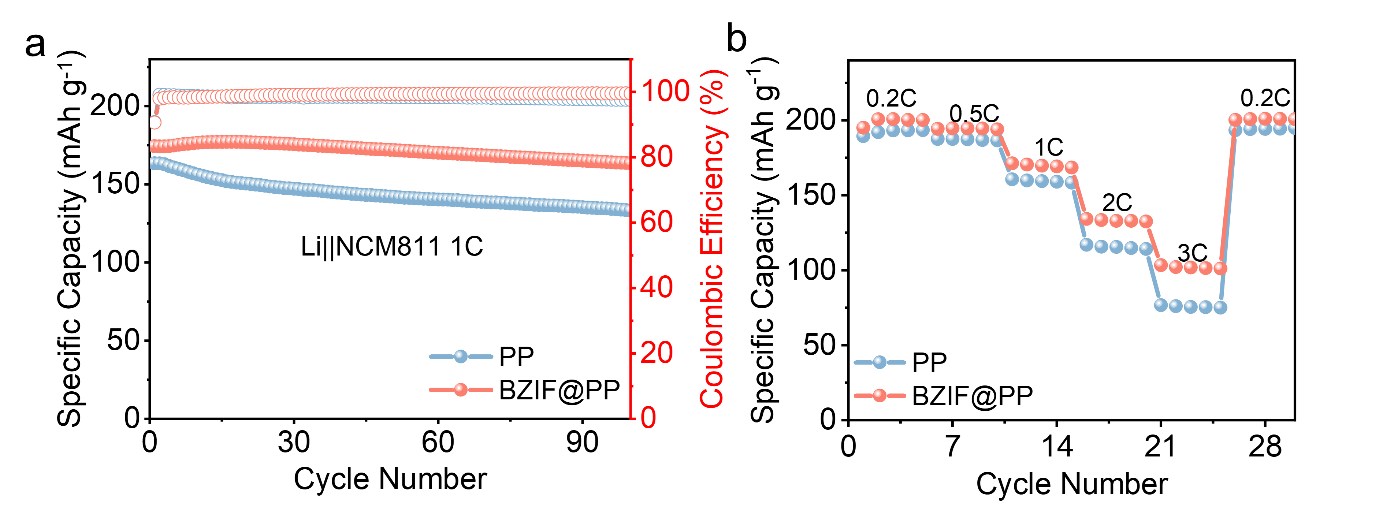
**

**Figure S22.** (a) Cycling performance at 1 C and (b) rate performance of Li||NCM811 cells with PP and BZIF@PP separators.
